# Recent Advances in Monocomponent Visible Light Photoinitiating Systems Based on Sulfonium Salts

**DOI:** 10.3390/polym15214202

**Published:** 2023-10-24

**Authors:** Frédéric Dumur

**Affiliations:** Aix Marseille Univ, CNRS, ICR, UMR 7273, F-13397 Marseille, France; frederic.dumur@univ-amu.fr

**Keywords:** photoinitiator, visible light, photopolymerization, LED, sulfonium, monocomponent, photoacid generator

## Abstract

During the last decades, multicomponent photoinitiating systems have been the focus of intense research efforts, especially for the design of visible light photoinitiating systems. Although highly reactive three-component and even four-component photoinitiating systems have been designed, the complexity to elaborate such mixtures has incited researchers to design monocomponent Type II photoinitiators. Using this approach, the photosensitizer and the radical/cation generator can be combined within a unique molecule, greatly simplifying the elaboration of the photocurable resins. In this field, sulfonium salts are remarkable photoinitiators but these structures lack absorption in the visible range. Over the years, various structural modifications have been carried out in order to redshift their absorptions in the visible region. In this work, an overview of the different sulfonium salts activable under visible light and reported to date is proposed.

## 1. Introduction

**Figure 1:**
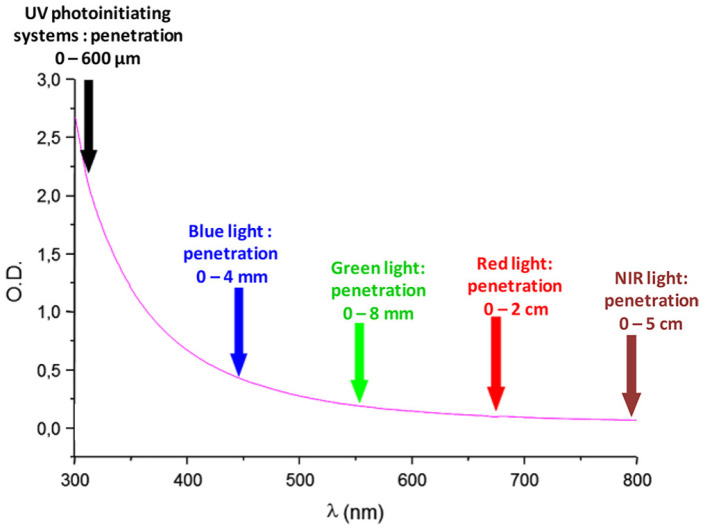
Penetration of light determined for a polystyrene latex (average diameter around 112 nm). Reprinted with permission from Ref. [143].

In recent years, photopolymerization has been the subject of intense research efforts with regards to the numerous applications making use of this polymerization technique [1,2,3,4,5,6,7,8,9,10,11,12,13,14,15]. Among them, applications such as solvent-free paints, adhesives, coatings and varnishes, dental restoration materials, 3D and 4D printing or microelectronics can be cited as relevant examples [9,10,16,17,18,19,20,21,22,23,24,25,26]. With the aim of designing efficient photoinitiating systems, multicomponent systems have been extensively investigated, especially for the design of visible light photoinitiating systems. Indeed, although historically photopolymerization was carried out in the UV range, this approach is nowadays more and more contested due to safety concerns related to UV light (skin cancers and eye damages) [27,28]. Parallel to this, energy sobriety imposed by numerous countries is an important parameter to consider and UV irradiation setups are, most of the time, energy-consuming devices which do not fit with the current energy efficiency action plan proposed by numerous countries. To date, since 2020, Hg lamps cannot be commercialized anymore, resulting from the treaty adopted in 2013 by numerous countries and resulting from the United Nations Environmental Program (UNEP) Minamata Convention on Mercury. Disinterest for UV photoinitiating systems is also related to the higher light penetration in resins that is achievable upon irradiation in the visible range. As shown in Figure 1, it can vary from a few millimeters up to a few centimeters depending on the irradiation wavelength. If this approach is of interest, photons of the visible spectrum exhibit lower energy than UV photons so that visible light photoinitiating systems of higher reactivity should be designed in order to compensate for this reduction of energy. To address this issue, a wide range of structures have been explored as exemplified by camphorquinone [29,30], thiophenes [31], push-pull dyes [2,32,33], anthracenes [34], acridine-1,8-diones [35], benzylidene ketones [36,37,38,39,40,41], chromones and flavones [42,43,44], perylenes [45], diketopyrrolopyrroles [46,47], naphthalimides [48,49,50,51,52,53,54], cyclohexanones [55,56,57,58], chalcones [24,59,60,61,62,63,64], dithienophosphole derivatives [65], thioxanthones [66,67,68,69,70,71,72,73,74,75,76,77,78,79,80], benzophenones [81,82,83,84], cyanines [85], bodipy [66,86,87,88,89,90], NIR dyes [91], anthraquinones [92], curcumin [93,94,95,96], furan derivatives [97], pyrenes [98,99,100,101], iodonium salts [66,102,103,104,105,106,107], carbazoles [108,109,110,111,112,113,114], quinoxalines [115,116,117,118,119,120,121,122,123,124,125,126,127,128], glyoxylates [129], helicenes [130], photochroms [131] and naphthoquinones [132]. Metal complexes were also investigated such as copper complexes [133,134,135], zinc complexes [136], gold complexes [137], iridium complexes [138,139,140] or iron complexes [141,142].

**Scheme 1:**
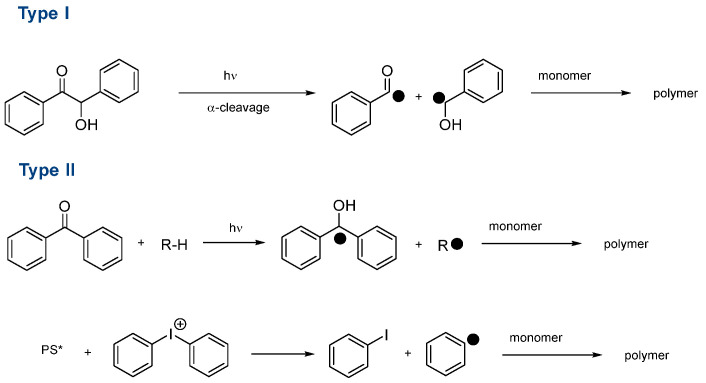
Comparison between Type I and Type II photoinitiators for radical generation.

Interestingly, highly efficient photoinitiating systems could be designed with these different chromophores using multicomponent systems. Indeed, as shown in Scheme 1, Type II photoinitiators can interact with hydrogen donors or electron donors/acceptors to generate reactive species. Over the years, reactivity of Type II photoinitiators could be improved by designing multicomponent systems, and four-component photoinitiating systems were even reported in the literature [144]. Considering the complexity of elaborating such photocurable resins, the design of monocomponent systems and especially of Type I photoinitiators is nowadays more and more studied. Among these structures, benzylketals, *o*-acyl-α-oximino ketones, trichloromethyl-*S*-triazine glyoxylates, acyloximino esters, hydroxyacetophenones, α-haloacetophenones, hexaaryl biimidazoles (HABIs), benzoin derivatives, oxime esters, phosphine oxides or α-aminoalkylacetophenones were examined for their photoinitiating abilities [145,146,147]. As for specificities, the working principle of Type I photoinitiators is based on the selective cleavage of a specific bond that can homolytically cleave upon photoexcitation. But monocomponent systems can also be developed with Type II photoinitiators. This strategy was notably successfully when applied to the design of monocomponent systems based on diaryliodonium salts [66,148,149,150]. By combining the photosensitizer and the iodonium salt within a unique molecule, efficient photoinitiating systems could be developed. In the field of onium salts, diaryliodonium salts were not the only structures studied for the design of monocomponent systems, and sulfonium salts were also identified as promising candidates [151,152].

**Scheme 2:**
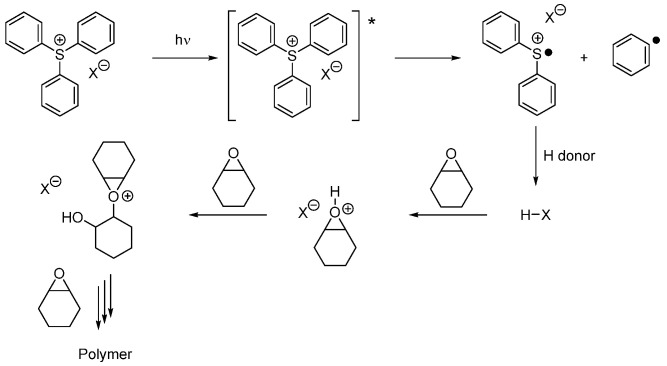
Mechanism of cationic polymerization of epoxides using triarylsulfonium salts. (* corresponds to the excited state).

Historically, diaryliodonium salts and sulfonium salts were studied starting from the 1970s by Crivello and coworkers for the cationic polymerization of epoxides [103,153,154,155,156,157]. Upon light irradiation, triarylsulfonium salts can release Brönsted acids. However, photosensitivity of triarylsulfonium is centred in the far UV region, rendering their photoactivation with visible light impossible. Sulfonium salts also suffer from a low solubility in numerous monomers, rendering these salts less attractive than iodonium salts for industrial applications [158]. Photoacid generation also strongly depends on the counter-ions used to prepare the salts and the following order of reactivity (SbF_6_^−^ > AsF_6_^−^ > PF_6_^−^ ≫ BF_4_^−^) could be determined [159]. Recently, new counter-anions were examined such as the tetrakis(perfluoro-*tert*-butyloxy)aluminate anion or the *tris*((trifluoromethyl) sulfonyl)methanide anion that makes sulfonium salts more reactive than those prepared with hexafluoroantimonate (SbF_6_^−^) [160]. By using anions such as 2,2-difluoro-2-sulfoethyl tricyclo[3.3.1.13,7]decane-1-carboxylate or 1,1,2,2,3,3,4,4,4-nonafluoro-1-butanesulfonate anions, hydrophobic sulfonium salts could be prepared [161]. Sulfonium salts bearing perfluorinated tetraphenylborates were also designed as photoinitiators for 3D printing experiments performed at high temperatures which are also named hot lithography [162]. The general initiation mechanism proposed for the cationic polymerization (CP) of epoxides upon irradiation is depicted in Scheme 2 [154,163,164,165]. Upon photoexcitation, an aryl radical and a radical cation can form. In the presence of a hydrogen donor, Bronsted acid can be generated, enabling the promotion of the ring-opening polymerization of epoxides.

Noticeably, sulfonium salts are versatile structures since protons and free radicals can be both formed, enabling the initiation of cationic and free radical polymerization processes [166,167]. Sulfonium salts are also thermally stable structures, facilitating their storage. However, in 1995, an allylic sulfonium salt was reported by Yagci and coworkers as exhibiting a dual thermal and photochemical curing mode [168,169]. But, this dual initiating ability remains limited to a few structures [170]. A few sulfonium salts by the convenient substitution of their scaffolds could also act as latent thermal initiators and the polymerization of glycidyl phenyl ether could proceed efficiently at more than 80 °C and not at less than 60 °C [171]. Starting from the 1980s, numerous sulfonium salts were reported as efficient thermal initiators for the cationic polymerization of epoxides, but these structures were inefficient as photochemical initiators [172,173]. Recently, Allonas and coworkers developed a two-component system comprising a triarylsulfonium salt acting as a photoinitiator and a phenyldialkylsulfonium salt acting as a thermal initiator. By this unique combination, a photochemically induced thermal polymerization of epoxides could be initiated [159]. By the heat released by the photoinduced cationic polymerization of epoxides, thermal decomposition of the phenyldialkylsulfonium salts could be induced so that carbon-fiber-reinforced polymers with a concentration of carbon fibers up to 50 wt% could be prepared upon irradiation at 365 nm. If triarylsulfonium salts were popular photoinitiators, trialkylsulfonium salts were also studied, as exemplified with dimethylphenacylsulfonium salts bearing various borate anions [174]. As a drawback, due to the lack of aromatic rings and therefore lack of chromophores on the sulfonium salts, visible light photoinitiating systems could only be developed by mean of the sensitization by various styryl dyes. Using the sensitization approach, photopolymerization experiments could be performed at 488, 514 and even 647 nm. Still, based on phenacyl sulfonium salts, Yagci and coworkers developed a diphenylphenacyl sulfonium salt exhibiting a dual photoinitiating ability for free radical and cationic polymerizations. Despite the presence of the diphenyl system, the absorption remained centered in the UV range, below 350 nm [175].

Over the years, two factors have been identified as being crucial in order to improve photoacid generation of sulfonium salts. First, the excited-state energy of the chromophore should be sufficiently high to initiate the photocleavage [176]. Indeed, the rate of the C–S bond cleavage was determined as being proportional to the energy gap existing between the LUMO (σ* orbital) of the sulfonium group and the LUMO + 1 (π* orbital) of the chromophore in the excited state [177]. Second, the radical cations and the neutral radicals formed after photolysis should be stable enough to avoid radical recombination [178]. Considering that benchmark sulfonium salts naturally absorb in the UV range [179], visible light photopolymerization can be initiated only if photosensitizers are used [180,181]. However, efficacy of the photoinduced electron transfer relies on a bimolecular process that is strongly influenced by the viscosity of the resin. In this context and to simplify the composition of resins, the design of monocomponent systems in which the photosensitizer can be directly connected to the sulfonium group was examined, offering a unique opportunity to design monocomponent systems. In this review, an overview of the different sulfonium salts activable under visible light is provided.

## 2. Sulfonium Salts Activable under Visible Light

### 2.1. Advantages, Disadvantages and Limitations

The design of monocomponent photoinitiating systems based on sulfonium salts and absorbing in the visible range is an interesting approach. Using this approach, an intramolecular sensitization process can occur. In addition, some advantages and limitations can be mentioned concerning the covalent linkage of a chromophore to a sulfonium salt. Notably, to redshift the absorption of salts exhibiting a UV-centered absorption is hard work. Nowadays, Type II photoinitiating systems that are proposed in the literature exhibit chromophores with structures totally disconnected from these historical UV photoinitiators (see the list of chromophores of innovative structures reported during the last 15 years). As a consequence of this, sulfonium salts with visible light absorption are costly and obtained in multistep syntheses. The benefits of intramolecular sensitization vs. the traditional intermolecular sensitization are sometimes low. A series of pros and cons for intramolecular vs. intermolecular sensitization is presented in Table 1.

### 2.2. 1,3,5-Triphenyl-2-Pyrazoline Derivatives

**Figure 2:**
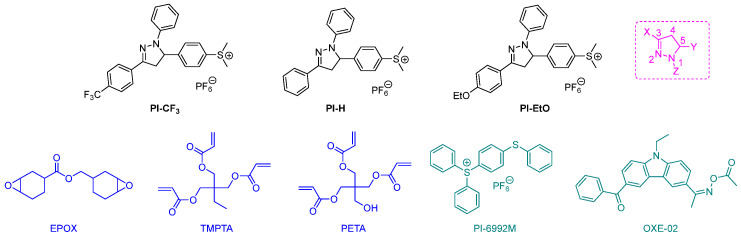
Chemical structures of 1,3,5-triphenyl-2-pyrazoline derivatives, different monomers and additives.

With the aim of providing photosensitivity in the visible region, a series of dimethylsulfonium salts derived from 1,3,5-triphenyl-2-pyrazoline derivatives were proposed in 2021 by Jin and coworkers as one-photon and two-photon photoinitiators (See Figure 2) [182]. The choice of 1,3,5-triphenyl-2-pyrazoline as the chromophore was notably motivated by the two-photon capability of this group. Indeed, in 2014, a two-photon absorption (TPA) cross-section of 280 GM was notably reported by Mysliwiec and coworkers for a 1,3,5-triphenyl-2-pyrazoline derivative, constituting an excellent basis for the design of TPA photoinitiators [183]. Due to the intramolecular charge transfer existing between the nitrogen atom N1 and the carbon C3 of the pyrazoline ring (see Figure 2 for numbering), 1,3,5-triphenyl-2-pyrazolines also efficiently absorb in the visible range [184], and the presence of a tertiary nitrogen atom can also contribute to stabilizing the radical cations, improving the quantum yield of photoacid generation.

**Figure 3:**
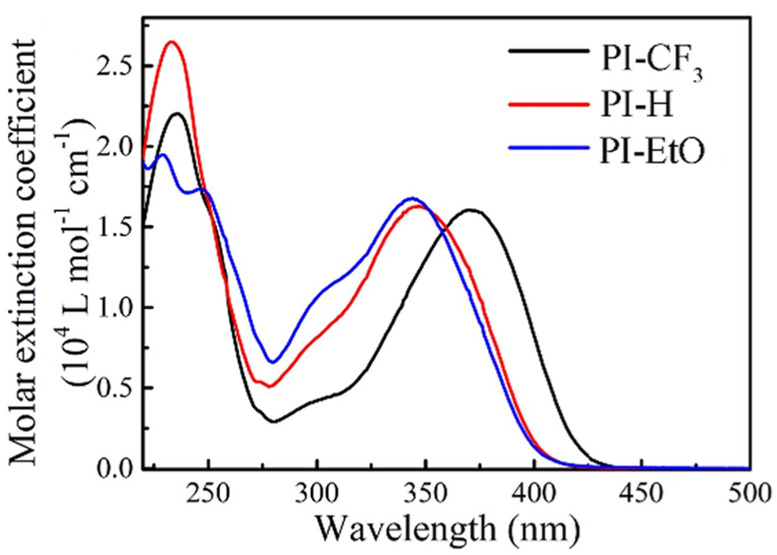
UV-visible absorption spectra of 1,3,5-triphenyl-2-pyrazoline derivatives in acetonitrile. Reproduced with permission of Ref. [182].

Depending on the substituent, absorption maxima varying between 344 nm for PI-EtO, 347 nm for PI-H and 372 nm for PI-CF3 could be determined in acetonitrile. The most redshifted absorption was found for PI-CF3 bearing a strong electron-donating group (see Figure 3).

**Figure 4:**
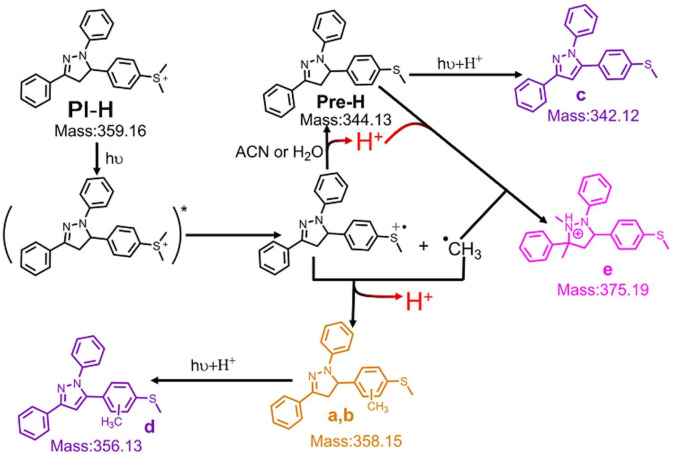
The different byproducts identified during the photolysis of PI-H in acetonitrile upon irradiation at 365 nm with an LED. Reproduced with permission of Ref. [182]. (* corresponds to the excited state).

Theoretical calculations revealed the S_0_–S_1_ and the S_0_–S_2_ transitions correspond to a π–σ* and a π–π* transition, respectively. Photolysis of PI-H performed upon irradiation at 365 nm with a LED revealed the photolysis process to be very fast. In particular, the reformation of the PI-H precursor, i.e., pre-H, was evidenced. However, it only corresponded to 20% of the photolysis products and the formation of numerous byproducts formed during photolysis were also detected. Indeed, the methyl radicals thus formed can attack the phenyl rings of pyrazolines, producing different byproducts (see Figure 4). This undesired reaction could be efficiently avoided in a solution by adding an excess of triethylamine [185].

Investigation of the quantum yields of photoacid generation at 365, 385, 395, 405 and 425 nm revealed the quantum yields decrease by irradiating at long wavelengths. In the case of the best photoacid generator, i.e., PI-EtO, the quantum yield decreased from 0.65 at 365 nm to only 0.20 at 425 nm. If PI-CF3 obtained the lowest quantum yield at 365 nm (ϕ_H+_ = 0.60), this compound could still maintain a quantum yield of 0.31 at 425 nm due to its redshifted absorption (See Table 2).

Considering that the efficiency of photoacid generation is strongly dependent on the energy gap between the lowest unoccupied molecular orbital (LUMO) level of the σ* orbital of the sulfonium salts and the LUMO + 1 level of the π* orbital of pyrazolines, these energy values were thus determined. If the LUMO level of sulfonium salts remained almost unchanged by modifying the substituent, conversely, the substitution pattern of the pyrazoline moiety drastically impacted the LUMO + 1 energy levels. Thus, ΔE(LUMO-LUMO + 1) varying between 0.54 eV for PI-EtO, 0.49 eV for PI-H and 0.25 eV for PI-CF3 were calculated, therefore perfectly fitting the quantum yields of photoacid generation. By cyclic voltammetry, the free energy change (ΔGet) could be determined using the Rehm–Weller equation [177,186,187,188,189,190]. Values varying between −21.73 kJ/mol for PI-CF3 and −55.82 kJ/mol for PI-EtO could be obtained. In particular, calculations revealed the electron transfer occurs from the singlet excited state, from the pyrazoline to the C–S σ* orbital, negative values of free energy changes being only obtained by considering the singlet excited state in the calculation and not the triplet state [178]. Photoinitiating abilities of the different sulfonium salts were examined during the cationic polymerization (CP) of (3,4-epoxycyclohexane)methyl-3,4-epoxycyclohexyl-carboxylate (EPOX) upon irradiation at different wavelengths with LEDs. As shown in Table 3, the order of reactivity determined for the quantum yields of photoacid generation was confirmed during the CP of EPOX, with PI-EtO outperforming the other sulfonium salts. Only at 425 nm, PI-CF3 could furnish a higher monomer conversion than PI-EtO and this is directly related to its redshifted absorption compared with that of PI-EtO. Thus, an EPOX conversion of 51.87% could be obtained with PI-CF3, higher than that obtained with PI-EtO (49.57%). Comparisons of the monomer conversion obtained with the benchmark photoinitiator PI-6992M and the different pyrazoline derivatives revealed PI-6992M to provide a higher conversion at 365 nm than the newly developed photoinitiators due to its higher molar extinction coefficient at this wavelength. At 385 nm, an opposite situation was found, resulting from its low molar extinction coefficient. Finally, in the visible range, no monomer conversion could be obtained with PI-6992M whereas the pyrazoline derivatives were still active, even at 425 nm.

Considering that methyl radicals are formed during photolysis, the free radical polymerization (FRP) of trimethylolpropane triacrylate (TMPTA) was also examined and comparisons of the photoinitiating abilities of PI-H and PI-CF3 were compared with that of OXE-02. As shown in Table 4, PI-CF3 and PI-H furnished lower monomer conversions at 365 and 385 nm than OXE-02, which exhibits better light absorption properties at these two wavelengths. However, at 395 and 405 nm, PI-CF3 and PI-H could outperform OXE-02. PI-H proved to be the best candidate for the FRP of TMPTA, the monomer conversion reaching 50.9% at 365 nm.

Finally, two pyrazoline derivatives were investigated as photoinitiators for two-photon polymerization. Two-photon absorption cross-sections of 105 and 75 GM were respectively determined for PI-CF3 and PI-H. A low threshold power was also determined during the two-photon microfabrication of structures under air using pentaerythritol triacrylate (PETA) as the monomer. During the FRP of PETA at 780 nm under air, a threshold value as low as 4.5 mW at 100–150 µm/s was obtained, evidencing the remarkable photoreactivity of these sulfonium salts.

### 2.3. Phenothiazine Derivatives

**Figure 5:**
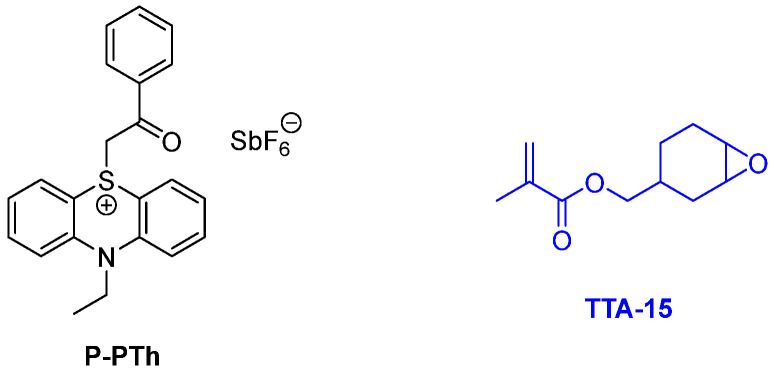
Chemical structures of P-PTh and TTA-15 used as bifunctional monomers.

**Figure 6:**
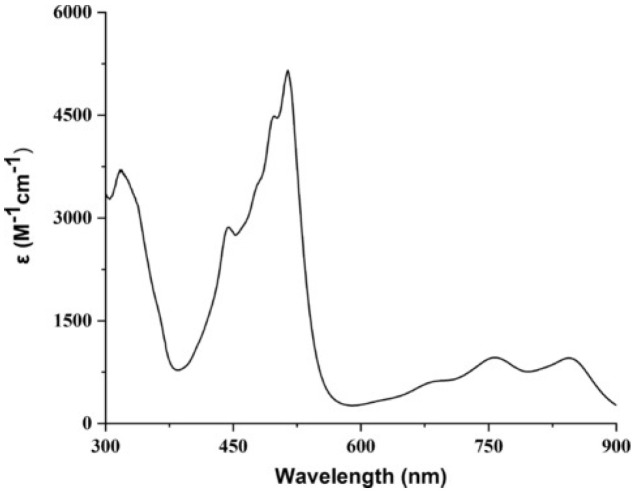
UV-visible absorption spectrum of P-PTh in acetonitrile. Reproduced with permission of Ref. [241].

Phenothiazine is an interesting scaffold for the design of visible light photoinitiators. Indeed, phenothiazine is a polyaromatic structure absorbing in the visible range, even without substituents, so that various Type I and Type II photoinitiators were designed and synthesized with this structure [191,192,193,194,195,196,197]. This is also an excellent electron donor that was extensively used for the design of push–pull dyes developed for different applications in organic electronics such as energy conversion [198,199,200,201,202,203,204,205,206,207,208,209,210], light-emitting materials for organic light-emitting diodes (OLEDs) [211,212,213,214,215,216,217,218,219,220,221,222,223] or semi-conductors for organic field effect transistors (OFETs) [199,224,225,226,227,228,229]. Phenothiazines were also developed as fluorescent probes for the detection of various molecules and ions in organic and aqueous solutions, food, live cells and animals [230,231,232,233,234,235,236,237,238,239,240]. In 2021, Yagci and coworkers proposed a phenacyl phenothiazinium salt (P-PTh) exhibiting a panchromatic absorption (see Figure 5) [241]. Indeed, as shown in Figure 6, the absorption spectrum of P-PTh in acetonitrile extends between 350 and 900 nm, with an absorption maximum at 514 nm.

**Scheme 3:**
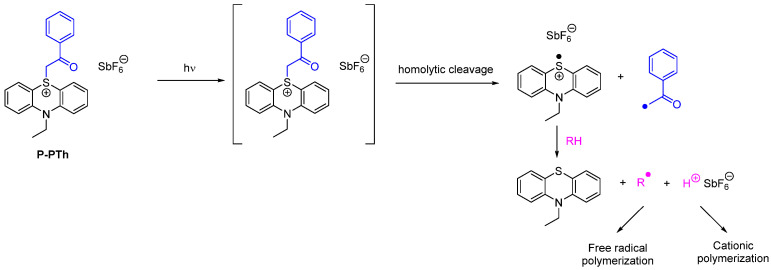
Photochemical mechanism involved in the decomposition of the phenacyl phenothiazinium salt (P-PTh).

Photolysis experiments performed in the solution and in the presence of an acid indicator revealed the release of Bronsted acid. By electron spin resonance (ESR), the formation of radicals resulting from the homolytic cleavage of the C–S bond was demonstrated, leading to the formation of phenacyl radicals and phenothiazine radical cations. In the presence of a hydrogen donor, Bronsted acid can be formed according to the mechanism depicted in Scheme 3. However, based on these experiments, the concomitant occurrence of a homolytic and heterolytic cleavage of the sulfonium salt cannot be ruled out. Laser flash photolysis experiments and additional spectroscopic investigations evidenced the triplet excited state of phenothiazine to play a key role in the mechanism of fragmentation [242].

**Figure 7:**
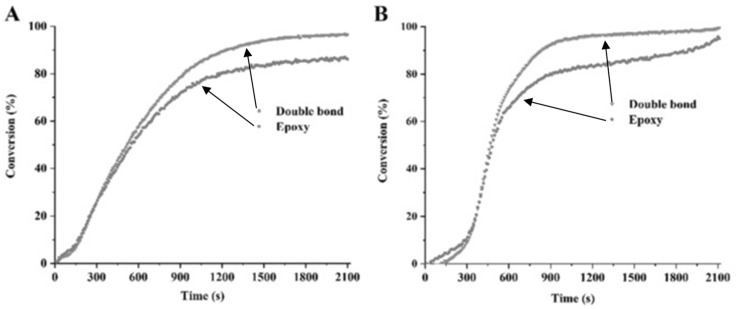
Polymerization profiles of TTA-15 upon irradiation at 365 nm (**A**) and 405 nm (**B**) with LEDs (I = 30 mW/cm^2^) [photoinitiator] = 1.72 × 10^−6^ mol/g resin. Reproduced with permission of Ref. [241].

Examination of the thermal stability of the phenacyl phenothiazinium salt (P-PTh) by TGA measurements revealed the decomposition temperature to be of 170 °C. P-PTh could also act as a thermal initiator, and onset polymerization temperatures of 100 °C in TMPTA and 160 °C in EPOX were respectively determined by DSC. However, upon storage of the resins at a moderate temperature, i.e., 60 °C in the dark for 100 days, a remarkable thermal stability could be determined, which constitutes an interesting feature for practical applications in industries. Polymerization tests performed at 365 and 405 nm revealed the EPOX conversions to be higher than 90% after 2100 s of irradiation with LEDs. A conversion of 60% could also be obtained at 850 nm, evidencing the versatile photoinitiating ability of P-PTh. Investigation of the FRP of TMPTA at 365 and 405 nm (30 mW/cm^2^) also furnished high monomer conversions, approximately 40% after 600 s of irradiation. This conversion could be greatly improved by using a broad-spectrum light source (260 mW/cm^2^) and a conversion of approximately 95% could be obtained for TMPTA. Due to the concomitant formation of radicals and cations, the polymerization of a bifunctional monomer, namely 7-oxabicyclo [4.1.0] heptan-3-yl) methyl methacrylate (TTA-15) was examined both at 365 and 405 nm. As shown in Figure 7, hybrid polymerization of TTA-15 furnished a rapid conversion of both functions, evidencing the FRP process to be independent from the CP.

### 2.4. Triphenylamine-Based Sulfonium Salts

**Figure 8:**
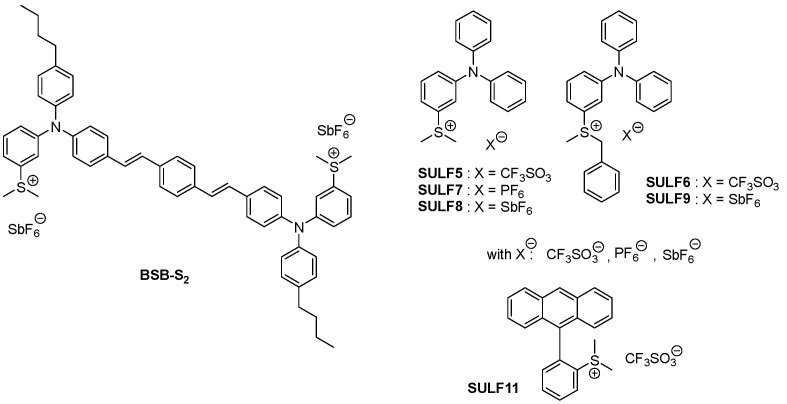
Different sulfonium salts based on triphenylamines as the chromophores.

Triphenylamine is an interesting scaffold for the design of visible light photoinitiators due to its excellent light absorption properties in the near-UV/visible range [243,244]. Dyes of high molar extinction coefficients can also be designed with this group [245,246,247,248]. The first triphenylamine-based sulfonium salt used as a two-photon-activatable photoacid generator BSB-S2 was reported as recent as 2002 by Marder and coworkers (see Figure 8) [249].

**Scheme 4:**
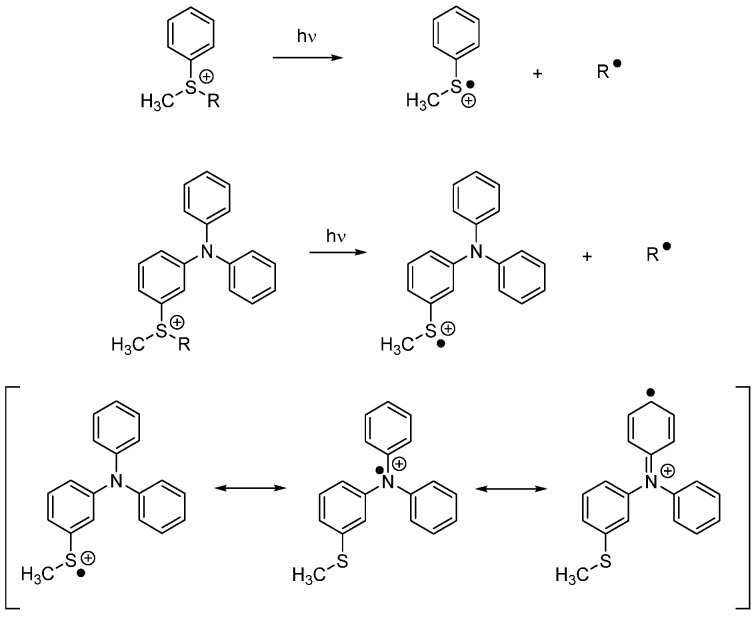
Interest of triphenylamine for the design of sulfonium salts.

Interestingly, BSB-S2 exhibited a large two-photon absorption cross-section of 690 GM and a quantum yield of photoacid generation of 0.5. The choice of triphenylamine as the chromophore was also justified by the fact that triaryl radical cations are relatively stable and can favor the C–S bond cleavage. Protonated forms of triarylamines are also strong acid (pKa < −5) so that the amino group cannot inhibit the cationic polymerization of epoxides. To end, the radical cation formed upon homolysis of the carbon-sulfur bond and centered on the triphenylamine moiety can be efficiently stabilized by mesomeric forms, as shown in Scheme 4.

**Figure 9:**
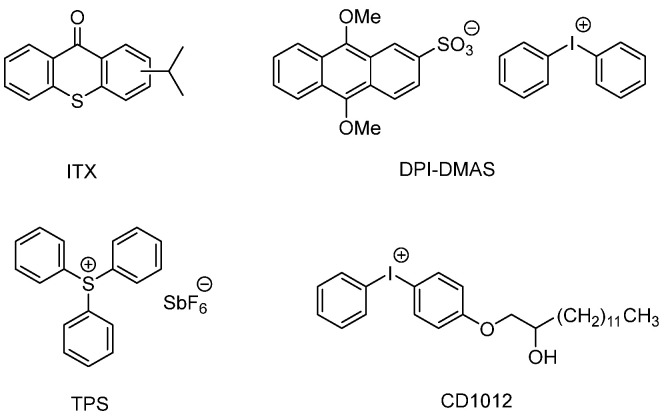
Chemical structures of benchmark two-photon initiators.

From the absorption viewpoint, the absorption of BSB-S2 was broad, extending between 250 and 450 nm. Two-photon microstructures could be obtained by polymerizing epoxides at 745 nm. Due to the presence of the triphenylamine moieties in BSB-S2, a low polymerization threshold power of 2.4 mW at 710 nm was determined during the CP of the Araldite CY179MA epoxide resin (composed of a mixture of 7-oxabicyclo[4.1.0]heptane-3-carboxylic acid and 7-oxabicyclo[4.1.0]hept-3-ylmethyl ester). This value is greatly lower than that determined for benchmark photoinitiators such as [4-[(2-hydroxytetradecyl) oxy]phenyl]phenyliodonium hexafluoroantimonate (CD1012), isopropylthioxanthone (ITX), diphenyliodonium 9,10-dimethoxyanthracene sulfonate (DPI-DMAS) or triphenylsulfonium hexafluoroantimonate (TPS) (see Figure 9 and Table 5) [250]. BSB-S2 was also an excellent candidate for a chemically amplified resist system designed for two-photon lithography [251].

In the same year, the same authors examined a series of sulfonium salts (SULF5-SULF9) exhibiting near-UV/visible absorption in order to investigate their photophysical properties (see Figure 8) [178]. Interestingly, by varying the counter-anion (CF_3_SO_3_^−^, PF_6_^−^, SbF_6_^−^) no modification of the quantum yields of photoacid generation was detected and a value of ca. 0.5 was determined for all salts. This is directly related to the fact that the different sulfonium salts exhibit identical absorption spectra due to the presence of the same chromophore. Photolysis experiments of the different sulfonium salts carried out at 294 nm enabled the identification of several photolysis products. Among them, formation of 3-methylthiotriphenylamine (52% of the sample), methyl-substituted 3-methylthiotriphenylamine (10% of the sample) and two products comprising a carbazole moiety and resulting from an intramolecular coupling (18% of the sample) could be identified as the four main products of the photolysis. Compared with triarylsulfonium salts (ϕ_H+_ = 0.53), photoacid generation of SULF-5-SULF9 was not adversely affected by the presence of the triphenylamine moiety, and quantum yields of photoacid generation of 0.48, 0.48, 0.47 and 0.41 were determined for SULF5 and SULF7-SULF9, respectively, using the method reported by Scaiano and coworkers [252]. These results are better than those obtained for the phenyl dimethylsulfonium salt for which no photoacid generation was detected in the same conditions [177]. In this case, a competition between the C–S bond cleavage and the radiative decay from the singlet excited state was suggested as the main reason impeding photoacid generation with this sulfonium salt. However, these photoacid quantum yields are comparable to those obtained for a polyaromatic structure such as the anthracenyl sulfonium salt SULF11 (see Figure 8). In this case, the rate of photocleavage of the C–S bond is higher than that of the fluorescence decay, enabling efficient photoacid generation [176]. Determination of the free energy change (ΔGet) using the Rehm–Weller equation revealed the photoinduced electron transfer from the aromatic amine to the low-lying C–S σ* orbital occurs from the first excited singlet state, for all investigated structures, as previously evidenced by Yagci and coworkers for a phenothiazine-substituted sulfonium salt [241]. As observed for the triphenylamine derivatives, the anthracenyl sulfonium salt SULF11 benefits from the electron-donating ability of the polyaromatic group, activating the dissociation of the S–C bond by stabilizing the resulting anthracenyl methyl sulfide radical cation formed by homolytic cleavage. If the choice of the counter-anion was without influence for photoacid generation, a different trend was found during the photopolymerization experiments. Indeed, polymerization efficiencies of the different sulfonium salts were strongly dependent on the counter-ion. As previously shown in the literature, nucleophilicity of the anion can strongly impact the strength of the ion pair formed by the anion and the cation so that the reactivity of the cation in photopolymerization can be affected. In previous works, the following scale of reactivity depending on the counter-anions used was established for iodonium salts: BF_4_^−^ < PF_6_^−^ < AsF_6_^−^ < SbF_6_^−^ < B(C_6_F_5_)_4_^−^ < Ga(C_6_F_5_)_4_^−^ [253]. In the present case, the role of the anion on the polymerization efficiency of the sulfonium salts was clearly evidenced and determined as being comparable to that observed for iodonium salts. Thus, if no polymerization of cyclohexene oxide (CHO) could be detected with SULF5 even after one hour of irradiation at 300 nm, 90% conversions could be obtained within 130 s with SULF8 and SULF9. In addition, a higher polymerization rate was determined for SULF9. To support these experimental results, the benzyl-substituted sulfonium salt SULF9 was determined as undergoing a heterolytic bond cleavage of the benzylic bond in the excited state in addition to the electron-transfer-mediated photoacid generation observed for SULF8, yielding additional benzylic cations that constitute another source of cations and thus can initiate the CP of CHO more efficiently [254].

**Figure 10:**
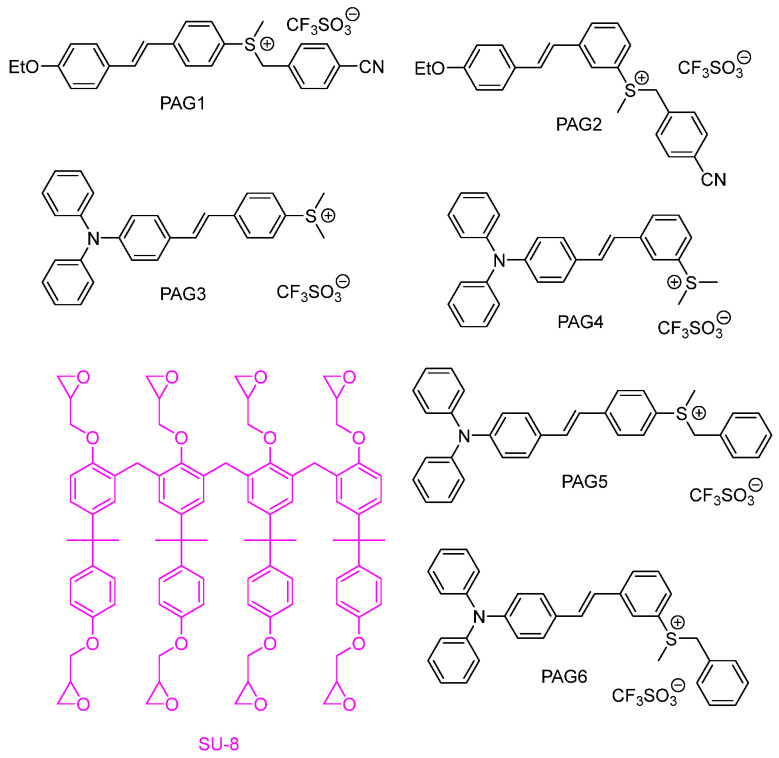
Examples of asymmetrically substituted sulfonium salts comprising a triphenylamine unit.

In 2002, BSB-S2 was reported as the first highly efficient photoacid generator activable in the near-infrared range and based on a symmetrical D–π–D structure. A two-photon sensitivity of 345 GM was measured at 710 nm, constituting a remarkable result. However, numerous works have also demonstrated that the extension of the π-conjugation of the chromophore could help to increase the two-photon sensitivity but also adversely affect the quantum yield of acid generation. This point was notably extensively studied by Belfield and coworkers [177,255,256,257]. In 2013, Malval and coworkers developed a series of asymmetrically substituted sulfonium salts PAG3-PAG6 on the basis of a D–π–A structure (see Figure 10) [258]. The different structures could act as efficient photoacid generators by one-photon excitation at 405 nm or by two-photon excitation at 800 nm. In order to tune the photoreactivity of the sulfonium salts, the influence of the *para*-to-*meta* substitution was examined. A comparison between dimethylsulfonium salts and methyl benzyl sulfonium salts was also carried out, previous works evidencing that benzyl cations could participate in the polymerization process.

From the absorption viewpoint, the presence of the triphenylamine group acting as an electron donor contributed to the redshift of the absorptions of PAG3-PAG6 compared with that of PAG1 and PAG2 bearing a weaker electron donor, i.e., an ethoxy group. Thus, if absorption maxima located at 346 and 324 nm were determined for PAG1 and PAG2, these values increased to 395 and 400 nm for PAG3 and PAG5, respectively. A redshift of the absorption by ca 20 nm was also determined for all the *para*-substituted sulfonium salts compared with the *meta*-substituted ones, resulting from a better conjugation between the donor and the acceptor (See Table 6). Absorption spectra of all asymmetric sulfonium salts were characterized by two absorption bands corresponding to aryl π–π* transitions at high energy and a π–σ* transition at lower energy. Examination of their photoacid generation ability at 405 nm revealed all triphenylamine-substituted sulfonium salts to be better photoacid generators than the ethoxy-substituted salts, except PAG3. Examination of the photoluminescence properties of PAG1-PAG6 revealed PAG3 to exhibit a photoluminescence quantum yield of 0.16 whereas values lower than 0.01 were found for all the other sulfonium salts. It was thus concluded that the excellent photoacid generation abilities of PAG4-PAG6 could be assigned to an efficient electron transfer from the triphenylamino group towards the sulfonium salt, resulting in an efficient quenching of luminescence. On the other hand, for PAG3, photoluminescence adversely competes with the photoinduced electron transfer, resulting in a low photoacid generation ability. Comparison of the photoacid generation ability of PAG3 and PAG4 with that of PAG5 and PAG6 revealed that PAG5 and PAG6 exhibit higher quantum yields of photoacid generation, attributable to the presence of better leaving groups, namely benzyl groups compared with the methyl groups. Remarkably, all *meta*-substituted photoacids proved to be better photoacids than their *para*-substituted analogues, due to their blue-shifted absorptions compared with that of their *para*-substituted analogues. This trend is consistent with previous works demonstrating that a redshift of the absorption of photoacids was accompanied by a reduction of photoacid generation ability. Benefits of the *para*-to-*meta* substitution in the photoacid generation of sulfonium salts was notably demonstrated with PAG1 and PAG2 [259]. In this case, an increase in the photoacid generation quantum yield by a factor 2.4 was determined. Among the most interesting findings, PAG3-PAG6 showed larger two-photon absorption cross-sections than PAG1 and PAG2. If values of 73 and 68 GM were determined for PAG1 and PAG2, respectively, these values increased up to 650 and 680 GM for PAG4 and PAG5.

**Figure 11:**
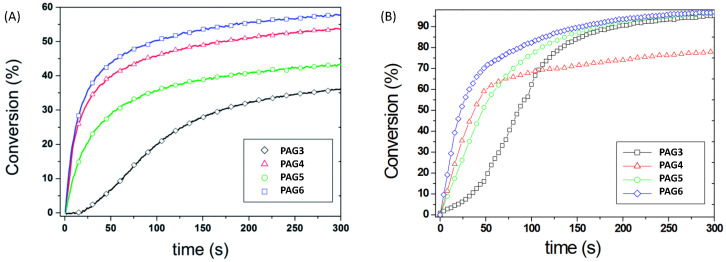
Photopolymerization profiles of EPOX (**A**) and CHO (**B**) upon irradiation at 405 nm (I = 5 mW/cm^2^) using 1 wt% photoacid generator. Reproduced with permission of Ref. [185].

The interest of these sulfonium salts was demonstrated during the cationic polymerization (CP) of (3,4-epoxycyclohexane)methyl-3,4-epoxycyclohexylcarboxylate (EPOX), and a diepoxide resin, i.e., SU-8 at 405 and 800 nm [185]. As shown in Figure 11, photopolymerization experiments performed at 405 nm (I = 5 mW/cm^2^) during the CP of EPOX confirmed the higher photoacid generation ability of the *meta*-substituted sulfonium salts compared with the *para*-substituted ones. Conversions of 58 and 52% could be respectively obtained with the *meta*-substituted salts PAG6 and PAG4 (1 wt%). Conversely, the *para*-substituted PAG3 furnished the lowest EPOX conversion, approximately 35% after 300 s of irradiation. These conversions could be greatly improved by using CHO as the monomer. In this case, conversions higher than 90% could be obtained with all photoacid generators except for PAG4, for which a conversion of 70% was determined.

Photopolymerization of the diepoxide resin SU-8 at 800 nm confirmed the trend established at 405 nm. Thus, high two-photon polymerization thresholds of 25 and 22 µJ were determined for PAG3 and PAG5, respectively, whereas these values decreased to only 10 µJ for the more reactive *meta*-substituted PAG4 and PAG6.

Finally, the high reactivity of PAG5 and PAG6 was demonstrated during the photopolymerization experiments performed under sunlight. Using CHO as the monomer, epoxide conversions higher than 80% could be determined after 20 min of sunlight exposure (I = 1.2 mW/cm^2^). However, it has to be noticed that this remarkable polymerization efficiency could only be obtained while increasing the photoinitiator content to 12 wt% and not 1 wt% with the artificial light sources.

**Figure 12:**
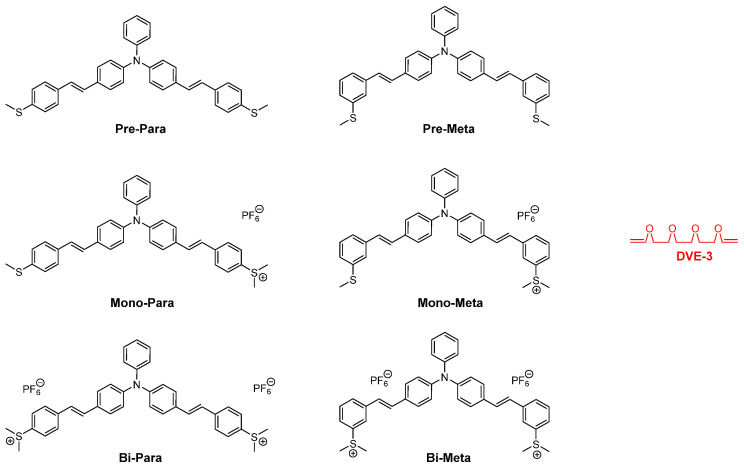
Chemical structures of mono and bifunctional sulfonium salts prepared by Malval and coworkers.

In the previous examples, only mono-sulfonium salts have been designed and synthesized. An extension of the former strategy was proposed in 2016 with the design of bifunctional sulfonium salts [260]. A comparison between mono and bifunctional salts could thus be established (see Figure 12).

**Figure 13:**
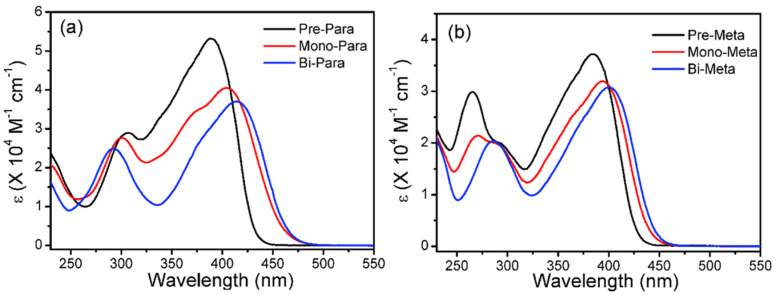
UV-visible absorption spectra of photoinitiators differing by the substitution pattern and recorded in acetonitrile. Reproduced with permission of Ref. [260] (**a**) UV-visible absorption of Pre-Para, Mono-Para, Bi-Para and (**b**) UV-visible absorption spectra of Pre-Meta, Mono-Meta and Bi-Meta.

**Figure 14:**
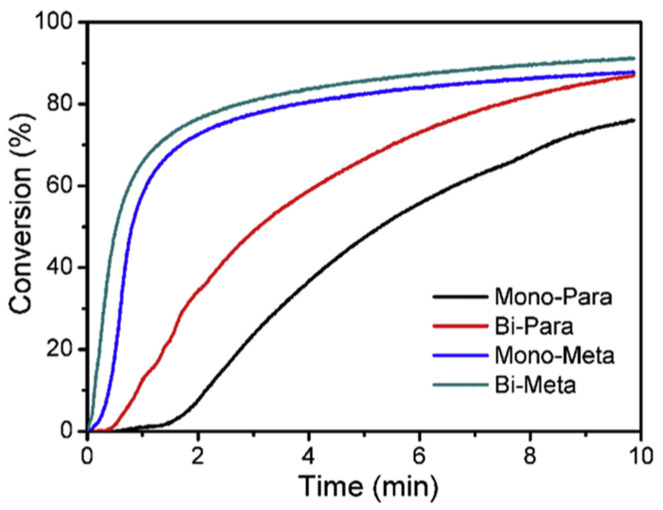
Polymerization profiles determined under air during the CP of DVE-3 by irradiating at 405 nm with a LED (5 mW/cm^2^), 1 wt% photoinitiator. Reproduced with permission of Ref. [260].

This strategy was pertinent since the di-substituted sulfonium salts showed photoacid generation abilities twice as high as that of their mono-substituted counterparts as well as improved two-photon absorption cross-sections. From the absorption viewpoint, the introduction of sulfonium groups redshifted the absorption of Mono-Para/Mono-Meta, Bi-Para/Bi-Meta compared with their parent structures Pre-Para/Pre-Meta (see Figure 13 and Table 7). Noticeably, this redshift was more important for the *para*-substituted sulfonium salts, attributable to a better conjugation of the sulfonium group with the rest of the structure. Benefits of the multi-branch approach were evidenced during the two-photon absorption measurements. Indeed, higher two-photon absorption cross-section (δ) values could be determined for the bifunctional PAGs compared with the monofunctional ones. Notably, the two-photon absorption cross-section δ value increased from 234 GM for Mono-Meta up to 745 GM for Bi-Meta. As anticipated during the design of these bifunctional sulfonium salts, the best photoacid generation efficiency was determined for Bi-Para and Bi-Meta, these sulfonium salts produce twice more Bronsted acids than the mono-substituted ones. Influence of the solvent used for photolysis was also demonstrated. Thus, higher photoacid generation efficiencies were determined in dichloromethane than in the more polar solvent, i.e., acetonitrile. Using triethyleneglycol divinyl ether (DVE-3) as the monomer, conversions higher than 90% could be determined with the four sulfonium salts upon irradiation at 405 nm. In this series of sulfonium salts, all the *meta*-substituted PAGs exhibited faster polymerization rates than the *para*-substituted ones. However, similar monomer conversions could be determined after 10 min of irradiation (see Figure 14).

This difference of reactivity was confirmed during the two-photon polymerization experiments performed at 780 nm using SU-8 as the resin.

**Figure 15:**
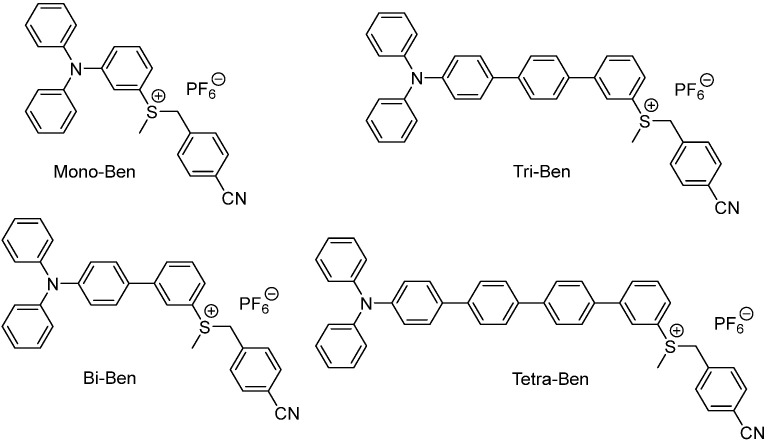
Chemical structures of oligomeric sulfonium salts.

In 2015, Malval and coworkers examined another strategy to extend the π-conjugation, and a series of oligomeric sulfonium salts differing by the number of phenyl rings used as the spacer between the triphenylamine and the sulfonium moieties were designed and synthesized (see Figure 15) [261]. 4-Cyanobenzyl groups were also introduced on the sulfonium moiety as photocleavable groups.

**Figure 16:**
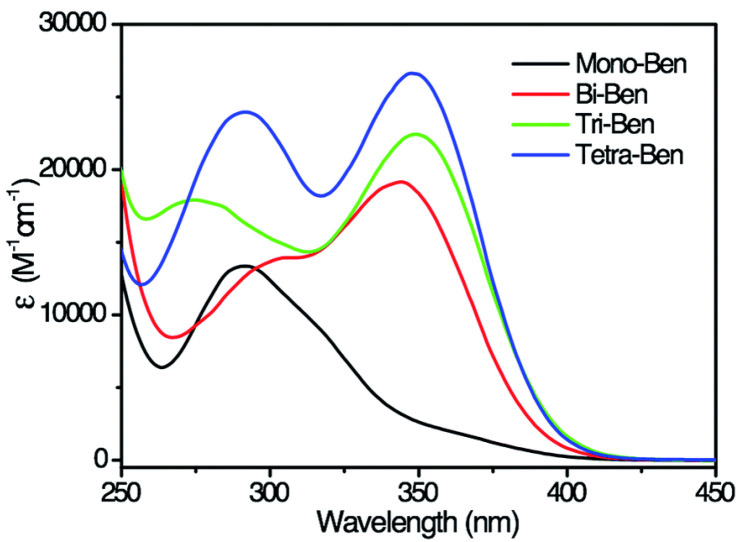
UV-visible absorption spectra of Mono-Ben, Bi-Ben, Tri-Ben and Tetra-Ben in acetonitrile. Reproduced with permission of Ref. [261].

Due to the presence of consecutive aromatic rings between the donor and the acceptor, the electronic delocalization was strongly limited so that the different sulfonium salts exhibited a strongly UV-centered absorption. If a significant redshift of the absorption maximum was found between Mono-Ben and Bi-Ben (292 and 344 nm, respectively), almost no additional redshift was detected for Tri-Ben and Tetra-Ben (349 and 351 nm, respectively), evidencing that the biarylic group was sufficient to interrupt the π-conjugation between the triphenylamine and the sulfonium moieties. An absorption extending up to 420 nm could be determined for Bi-Ben, Tri-Ben and Tetra-Ben, enabling the photopolymerization experiments to be performed under visible light (see Figure 16).

**Scheme 5:**
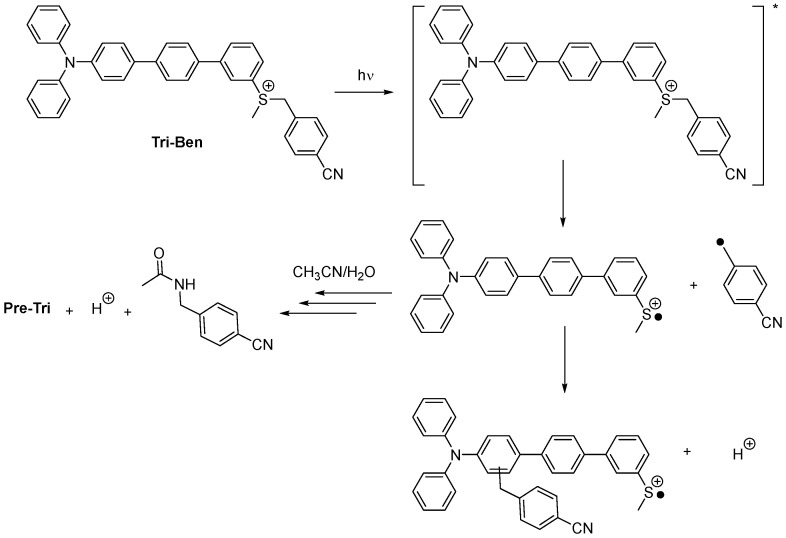
Mechanism of photoacid generation determined for oligomeric PAGs. (* correspond to the excited state).

Photoacid generation was examined at 365 nm in acetonitrile. The mechanism of photoacid generation is depicted in Scheme 5.

**Figure 17:**
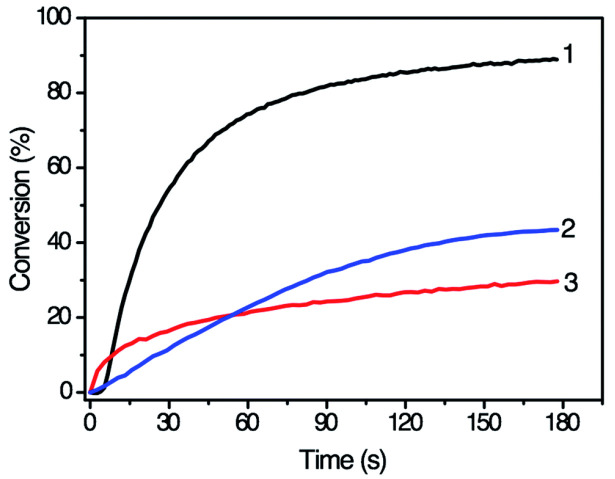
Photopolymerization profiles obtained with Tri-Ben as the photoinitiator: DVE-3 (1 wt% Tri-Ben, curve 1), TMPTA (Tri-Ben/MEDA, 1%/2%, *w*/*w*, curve 2) and EPOX (Tri-Ben/NVK, 1%/3%, *w*/*w*, curve 3) upon irradiation at 365 nm (15 mW/cm^2^). Reproduced with permission of Ref. [261].

In this series, photoacid generation ability increased from Mono-Ben to Bi-Ben and Tri-Ben (ϕ_H+_ = 0.60, 0.69 and 0.73, respectively) and then decreased for Tetra-Ben (ϕ_H+_ = 0.42). In the case of Mono-Ben, a higher photoacid generation ability than that of SULF9 (previously reported by Marder and coworkers) was demonstrated (ϕ_H+_ = 0.60 vs. 0.41 for SULF9), resulting from the substitution of the benzylic group by a cyano group in Mono-Ben [178]. This was assigned to the better leaving propensity of the 4-cyanobenzyl group, facilitating Bronsted acid generation. In the case of Tetra-Ben, a reduction of the photoacid generation ability was determined (ϕ_H+_ = 0.42). Furthermore, it remains greatly higher than that reported for anthracenyl and naphthacenyl sulfonium salts [176]. In terms of PAG design, it was thus concluded that the introduction of cyanobenzyl groups and benzene oligomers was beneficial for improving the photoacid generation ability. An optimal structure was thus identified with Tri-Ben. The superiority of Tri-Ben over the other PAGs was confirmed during the CP of EPOX and the FRP of TMPTA using 1 wt% photoinitiators (see Figure 17). Upon irradiation at 365 nm (I = 15 mW/cm^2^), conversions of 90, 40 and 30% were, respectively, determined for tri(ethylene glycol)divinyl ether (DVE-3), TMPTA and EPOX used as the monomers. Excellent results were also obtained during the CP of SU-8.

Examination of the thermal properties revealed the four derivatives to exhibit decomposition temperatures higher than 150 °C, which is sufficient for numerous practical applications. The different PAGs could also be used as two-photon initiators. Indeed, two-photon absorption cross-sections of 184, 230, 474 and 393 GM were determined at 700 nm. The highest two-photon absorption cross-section was determined for Tri-Ben which is therefore a versatile photoinitiator as it can efficiently initiate one-photon and two-photon polymerizations. By laser-scanning lithography (LSL) at 780 nm, the SU-8 resin containing 1 wt% Tri-Ben could efficiently polymerize and only near the focus of the laser beam. The 3D patterns exhibiting excellent spatial resolution could be obtained.

**Figure 18:**
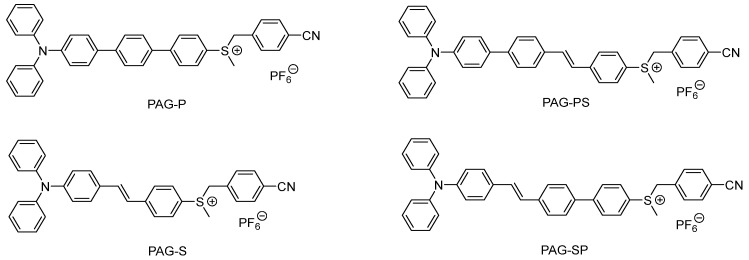
Chemical structures of different sulfonium salts combining double bonds and consecutive aromatic rings in their scaffolds.

Following this work, Malval and coworkers examined a series of *para*-substituted derivatives in which the position of the double bond as well as the number of consecutive aromatic rings were modified (see Figure 18) [262].

**Figure 19:**
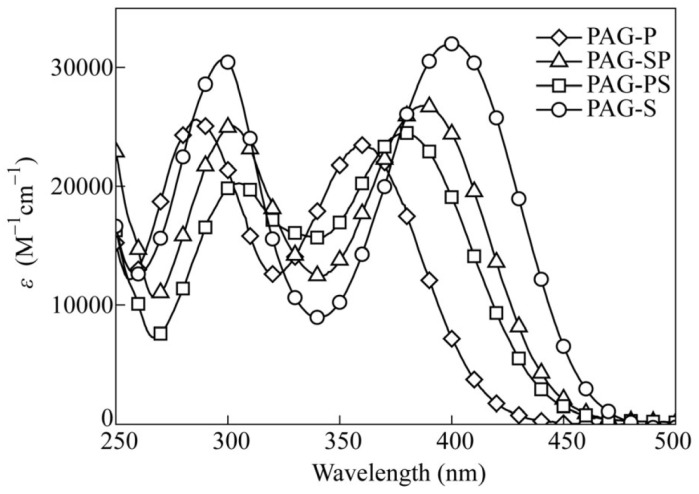
UV-visible absorption spectra of different sulfonium salts combining double bonds and consecutive aromatic rings in their scaffolds. Reproduced with permission of Ref. [262].

Compared with the previous *meta*-substituted series, a redshift of the absorption spectra was determined for all derivatives, consistent with a better conjugation between the donor and the acceptor. For the most conjugated structure, namely PAG-S, an absorption maximum peaking at 400 nm was determined in acetonitrile. Upon the addition of a biarylic system, a blueshift of the absorption maximum was determined for PAG-PS and PAG-SP, the highest blueshift being determined for PAG-PS (380 nm vs. 290 nm for PAG-SP). In the case of PAG-P, an absorption located at 360 nm was detected, resulting from the presence of the biarylic systems (see Figure 19). Logically, the redshift of the absorptions was accompanied by a reduction of the photoacid generation quantum yields. Thus, quantum yields of 0.58, 0.50, 0.48 and 0.32 were, respectively, determined for PAG-P, PAG-PS, PAG-SP and PAG-S, consistent with the trend previously determined for other sulfonium salts.

**Figure 20:**
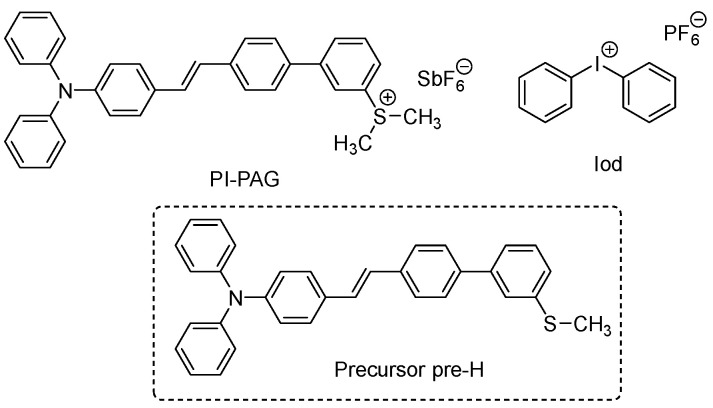
Sulfonium salt PI-PAG with the dual role of cationic initiator and photosensitizer for an iodonium salt (Iod). Chemical structure of precursor pre-H used for the synthesis of PI-PAG is also presented.

Photolysis of the sulfonium salts resulted in the generation of byproducts that often absorb at similar wavelengths than the parent sulfonium salt. Malval and coworkers advantageously used the absorption of these byproducts to induce the decomposition of an iodonium salt, namely diphenyliodonium hexafluorophosphate (Iod) (see Figure 20) [167]. In this aim, PI-PAG was designed and synthesized.

**Figure 21:**
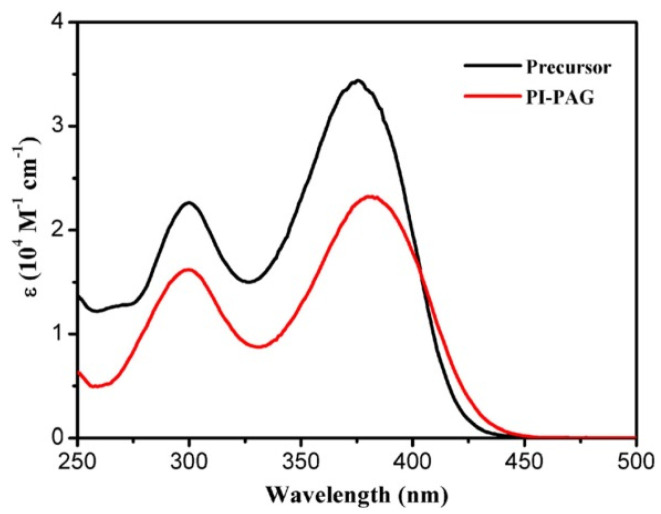
UV-visible absorption spectrum of PI-PAG and its corresponding precursor pre-H in acetonitrile. Reproduced with permission of Ref. [167].

Benefiting from an extended π-conjugated system but also from a biarylic system, an interesting absorption extending up to 450 nm could be obtained. A slight redshift of the absorption was obtained by converting the methylsulfanyl group in the precursor pre-H as a sulfonium group in PI-PAG (381 nm for PI-PAG vs. 374 nm for pre-H) (see Figure 21).

**Figure 22:**
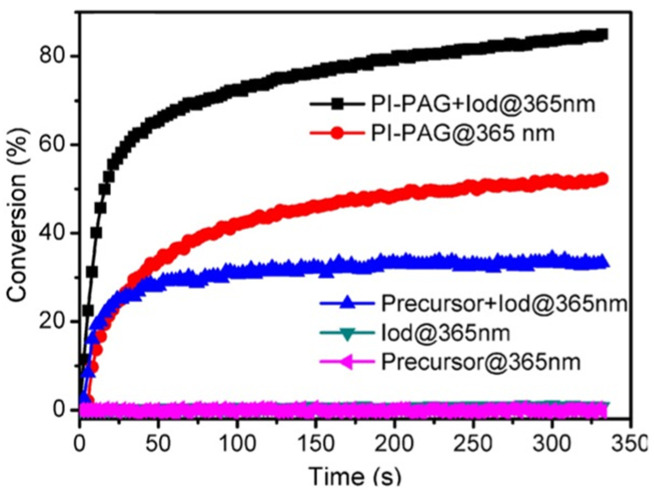
Photopolymerization profiles obtained during the ring-opening polymerization of EPOX under air upon irradiation at 365 nm (I = 40 mW/cm^2^). Iod (3 wt%), precursor pre-H (1 wt%), PI-PAG (1 wt%), PI-PAG/Iod (1%/3%, *w*/*w*) and precursor pre-H/Iod (1%/3%, *w*/*w*). Reproduced with permission of Ref. [167].

Photopolymerization experiments could thus be carried out at 365, 385, 405 and 425 nm. Upon irradiation at 385 nm, a quantum yield of photoacid generation of 0.44 could be determined for PI-PAG. Polymerization tests performed at 365 nm (I = 40 mW/cm^2^) in the presence of Iod revealed an improvement of the EPOX conversion from 52% with PI-PAG (1 wt%) up to 82% upon addition of Iod (3 wt%) (see Figure 22). It was thus concluded that the chromophore in PI-PAG could also sensitize the photodecomposition of Iod.

**Figure 23:**
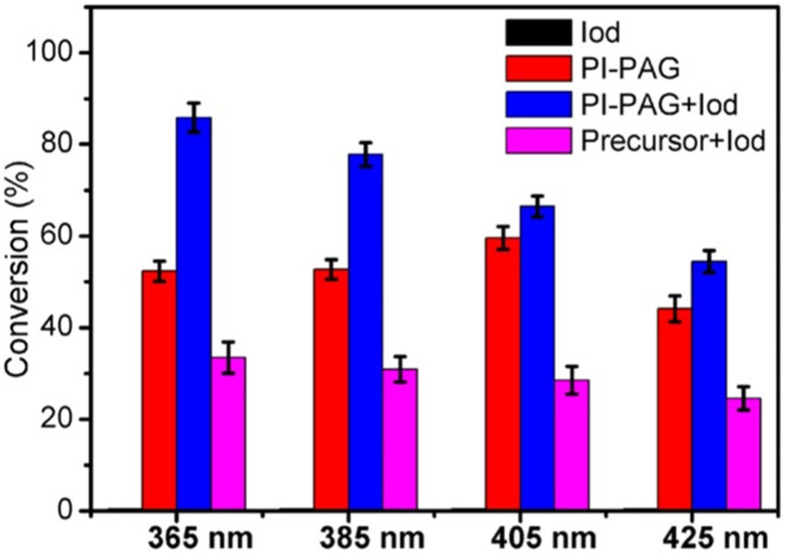
Monomer conversions determined during the CP of EPOX at different wavelengths using Iod (3 wt%), precursor pre-H (1 wt%), PI-PAG (1 wt%), PI-PAG/Iod (1%/3%, *w*/*w*) and precursor pre-H/Iod (1%/3%, *w*/*w*), I = 40 mW/cm^2^ for all LEDs. Reproduced with permission of Ref. [167].

This point was confirmed during the polymerization tests carried out with the precursor pre-H which was used for the synthesis of PI-PAG. Using the two-component precursor pre-H/Iod (1%/3%, *w*/*w*) system, a conversion of 30% could be determined after 300 s of irradiation, evidencing that precursor pre-H could act as a photosensitizer for Iod. Ability of precursor pre-H but also of the byproducts formed during the photolysis of PI-PAG to sensitize the decomposition of Iod was confirmed at 385, 405 and 425 nm. In these different cases, an improvement of the monomer conversion was still obtained with the two-component systems compared with PI-PAG used alone (see Figure 23). A reduction of the monomer conversion was detected at long wavelengths, consistent with a decrease in the molar extinction coefficients.

Photolysis experiments also revealed that the photolysis of PI-PAG ended within 5 s so that the contribution of the photolysis byproducts to sensitize Iod was clearly demonstrated. The following mechanism was suggested to support the improvement of monomer conversion in the presence of Iod (see Equations (1)–(6)). In this mechanism, the key step is the photoinduced electron transfer between the excited dye and the electron deficient Iod (2). Dye^•+^ is thus formed, constituting an initiating species. In the presence of the monomer, a hydrogen abstraction reaction can occur, generating Bronsted acid (see Equations (3) and (4)). Parallel to this, the phenyl radical Ph^•^ can also react with the monomer, generating M^+^.

Dye ⟶ Dye* (hν)(1)

Dye* + Ph_2_I^+^ ⟶ Dye^•+^ + Ph^•^ + Ph-I (2)

M-H + Dye^•+^ ⟶ Dye^+^-H + M^•^ (3)

Dye^+^-H ⟶ Dye + H^+^ (4)

M-H + Ph^•^ ⟶ Ph-H + M^•^ (5)

M^•^ + Ph_2_I^+^ ⟶ M^+^ + Ph-I + Ph^•^ (6)

Estimation of the Gibbs free energy change (ΔGet) using the Rehm-Weller equation revealed that the photoinduced electron transfer proceeds from the singlet excited state for both PI-PAG and precursor pre-H. Notably, in the case of the two-component precursor pre-H/Iod system, ΔGet = −180 kJ/mol was determined, constituting a high driving force for the photoinduced electron transfer. Occurrence of a photoinduced electron transfer from precursor pre-H to Iod was confirmed by fluorescence quenching measurements, with a complete quenching of the fluorescence of the precursor in the presence of Iod. In 2020, Yagci and coworkers revisited PI-PAG in the context of photopolymerization experiments carried out under low light intensities since the weak chemiluminescence of 9,10-diphenylanthracene (DPA) was used as an inner light source [263]. An emission centered at 430 nm could be obtained with DPA. Due to the weak light intensity, 20 min were required to obtain an efficient conversion of monomers such as *N*-vinyl carbazole (NVK) and *n*-butyl vinyl ether (BVE) bearing electron-donating moieties. In the case of cyclohexene oxide (CHO), 100 min were necessary to obtain acceptable monomer conversions. To support the high monomer conversions obtained with NVK and BVE, the concomitant occurrence of a cationic polymerization process issued from Bronsted acids and a free-radical-promoted cation polymerization process resulting from the oxidation of monomer-derived radicals by the sulfonium salt was suggested. Among the most interesting results, an efficient post-curing process could be evidenced. Thus, an increase in the CHO conversion from 20% could be obtained in the dark after 40 min after the irradiation has ceased. This is directly related to the formation of Bronsted acids that permit this efficient dark curing process.

**Figure 24:**
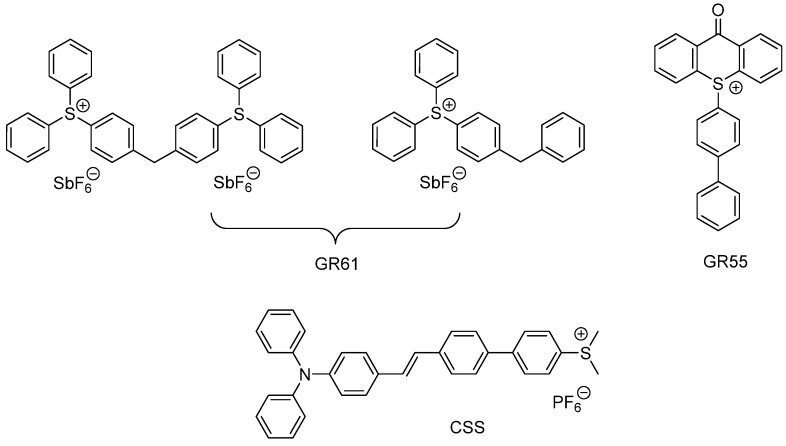
Chemical structures of sulfonium salts used during UCNPS-mediated photopolymerization.

In the same spirit, up-conversion nanoparticles (UCNPs) were recently used to activate sulfonium salts in the near-UV visible range while irradiating in the near-infrared range (see Figure 24) [264]. To date, only a few works have been reported in the literature concerning the use of UCNPs for developing safe polymerization processes [264,265,266,267,268,269,270,271]. Taking advantage of the simultaneous formation of Bronsted acids and radicals, hybrid polymerizations could be carried out, providing access to interpenetrated polymer networks (IPNs) [135,272,273,274,275,276,277,278]. Lanthanide-doped UCNPs β-NaYF_4_: 18% Yb, 0.5% Tm nanoparticles were used for up-conversion so that, upon irradiation at 980 nm, a blue light with emission peaks at 345 and 261 nm could be produced. Due to the better adequation of the absorptions of GR55 and CSS with that of the blue light, faster photolyses were obtained compared with GR61, for which a mismatch of the absorption was detected. Indeed, absorption of GR61 is strongly UV-centered and the use of a photosensitizer is commonly used to develop visible light photoinitiating systems with this mixture of sulfonium salts [279]. The high reactivity of GR55 was confirmed during the polymerization experiments, the best CHO conversion being obtained for sulfonium salt.

**Figure 25:**
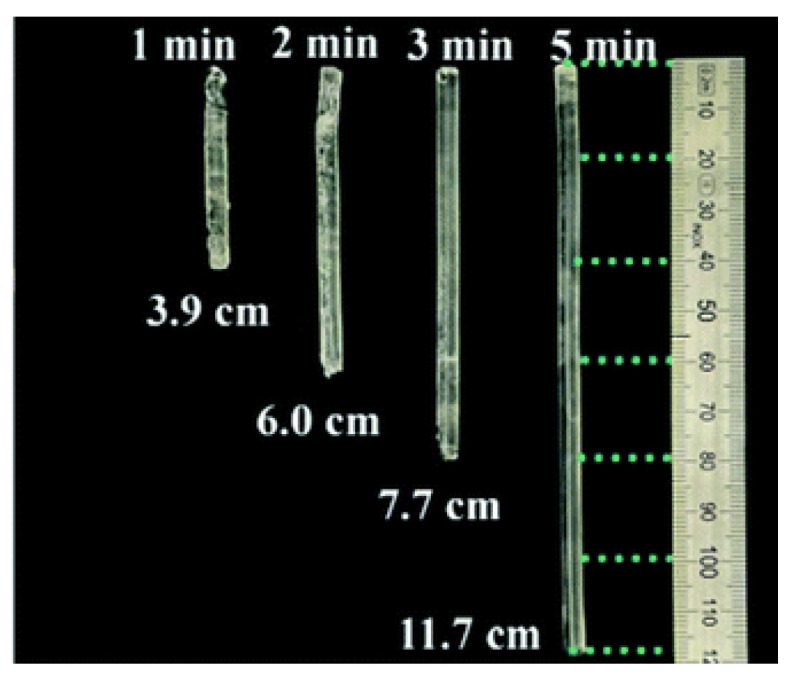
Curing depth determined during the hybrid polymerization of a TMPTA/EPOX blend upon irradiation of the samples at 980 nm with a laser (I = 11.6 W) for different irradiation times. Reproduced with permission of Ref. [264].

Here again, an important dark curing was evidenced since the CHO conversion increased from ca 20% after one hour in the dark, comparable to the results previously reported during the chemiluminescence-assisted cationic polymerization. Examination of the depth of the cure obtained during the radical/cationic hybrid polymerization of a TMPTA/EPOX (1/1) blend revealed that the curing depth reachs 3.9 cm after 2 min of irradiation at 980 nm (I = 11.6 W) and by using 0.4 wt% of UCNPs and 0.3 wt% of GR55. By elongating the irradiation time to 5 min, the remarkable curing depth of 11.7 cm could be evidenced (see Figure 25).

**Figure 26:**
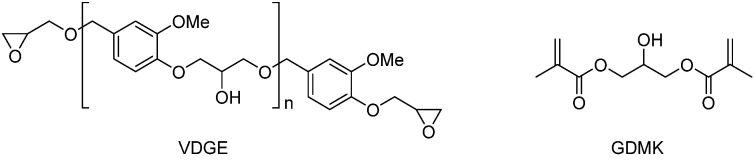
Chemical structures of VDGE and GDMK.

**Figure 27:**
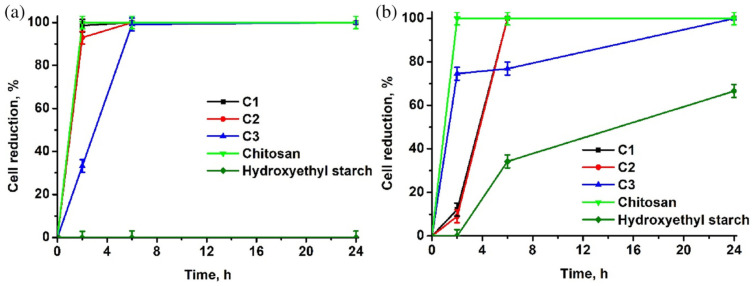
Reduction of *E. coli* (**a**) and *S. aureus* (**b**) cells during 24 h of contact time with films of vanillin-based polymers, chitosan and hydroxyethyl starch. Reproduced with permission of Ref. [280].

In 2022, the benchmark mixture of sulfonium salts, i.e., GR61, enabled the designing of antimicrobial coatings using a biobased epoxy monomer [280]. By increasing the vanillin alcohol diglycidyl ether (VDGE) content within the interpenetrated polymer networks prepared with glycerol dimethacrylate (GDMK), an improvement of the antimicrobial activities of the polymer films was determined (see Figure 26). Antibacterial and antifungal activities of the polymer blends comprising 66, 50 and 33% of VDGE were investigated by examining the viability of *E. coli* and *St. aureus* in contact with the polymer films. After 5 h, the viability of *E. coli* was reduced to 0% with all polymer films. In the case of *St. aureus*, 24 h of incubation were required to reduce the cell viability to 0%. In addition, as shown in Figure 27, the fastest reduction of *E. coli* and *St. aureus* cells were obtained for polymer films comprising 66 and 30% of VDGE. Comparison of the antibacterial activities of chitosan and hydroxyethyl starch with that of the newly developed sulfonium salts revealed the different sulfonium salts require longer exposure times than chitosan (approximately 24 h for a complete inhibition of the bacterial viability of *St. aureus* whereas two hours are only required with chitosan films). However, compared with hydroxyethyl starch, a better activity was found with the different sulfonium salts.

### 2.5. Bopidy-Based Sulfonium Salts

**Figure 28:**
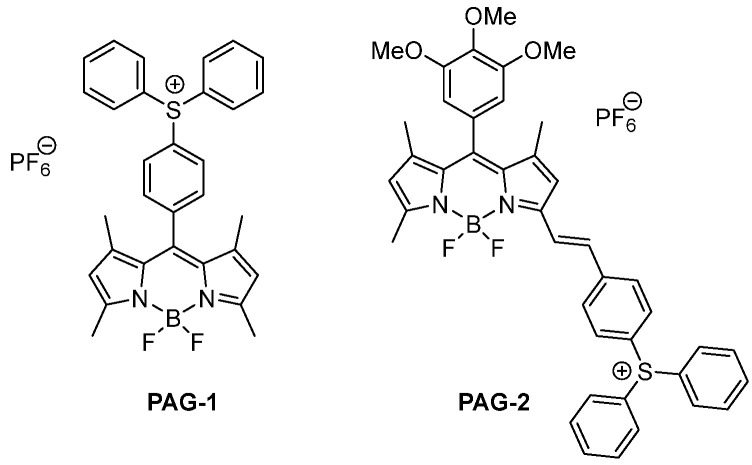
Chemical structures of bodipy-based sulfonium salts.

Bodipys, also named boron dipyrromethenes or 4,4-difluoro-4-bora-3a,4a-diaza-s-indecenes, have been identified, starting from 2000, as potential candidates for photoinitiation [281]. However, the introduction of the bodipy moiety onto sulfonium salts was only examined in 2020 by Zhang and coworkers that designed PAG-1 and PAG-2 (see Figure 28) [282]. The two sulfonium salts differing by the substitution pattern showed absorption maxima at 509 and 566 nm, respectively, in methanol. Due to the more extended structure of PAG-2 compared with that of PAG-1, a redshift of the absorption spectrum was logically detected. Noticeably, a low photoluminescence quantum yield was determined for the two salts, namely 0.02 for PAG-2 and 0.11 for PAG-1. Examination of their photoacid generation quantum yields revealed PAG-1 to be a better candidate since a yield of 7% was determined, higher than that obtained for PAG-2, namely 4%.

Polymerization experiments were carried out at 595 nm (I = 80 mW/cm^2^) using cyclohexane oxide (CHO) as the monomer and 0.8 wt% photoinitiators. The different experiments only revealed slow polymerization processes. After 30 min, 21% of conversion could only be determined consistent with their low photoacid generation abilities. Despite these low monomer conversions, these two structures can be cited as the sulfonium salts displaying the most redshifted absorption ever reported to date.

### 2.6. Charge Transfer Complexes

**Figure 29:**
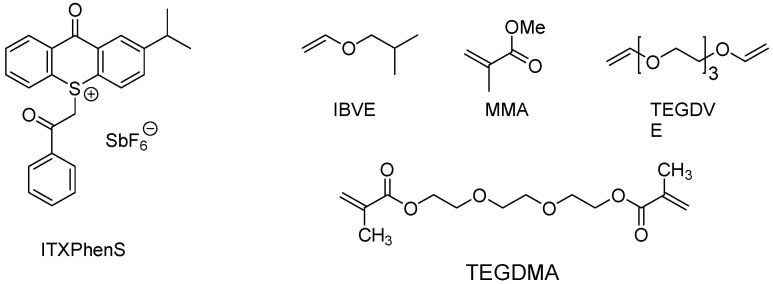
Chemical structures of ITXPhenS, different monomers and additives.

Sulfonium salts suffer from a UV-centered absorption so that these structures are inactive under visible light [159,283,284]. In order to extend their spectral activities, an interesting approach was proposed by Yagci and coworkers, consisting in generating charge transfer complexes (CTCs) [285,286]. The interest of the charge transfer approach relies on the fact that the combination of an electron donor and an electron acceptor not absorbing in the visible range can produce, once the CTC forms, a new species absorbing in the visible range. In 2019, an isopropylthioxanthone-based sulfonium salt (ITXPhenS) (see Figure 29) was notably examined as an electron-accepting group for an electron-rich amine, i.e., *N*,*N*-dimethylaniline (DMA), so that an interesting charge transfer complex (CTC) absorbing in the visible range could be produced [287].

**Figure 30:**
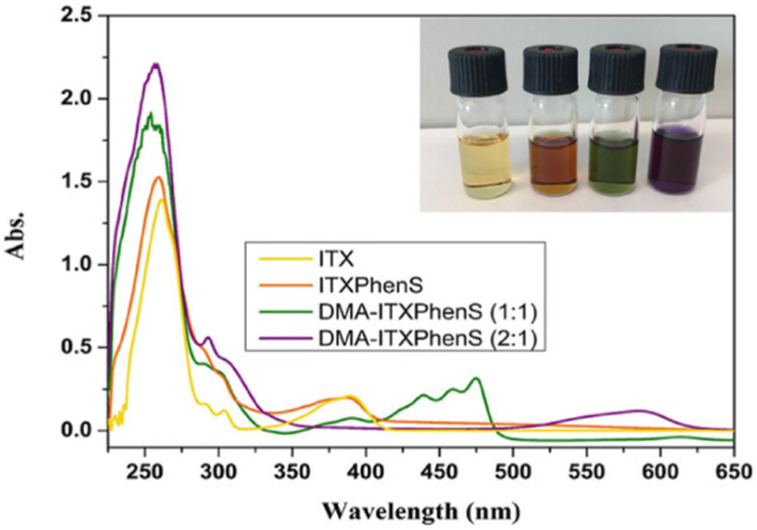
UV-visible absorption spectra of ITX, ITXPhenS and CTCs comprising different ITXPhenS/DMA ratios. Reproduced with permission of Ref. [287].

**Figure 31:**
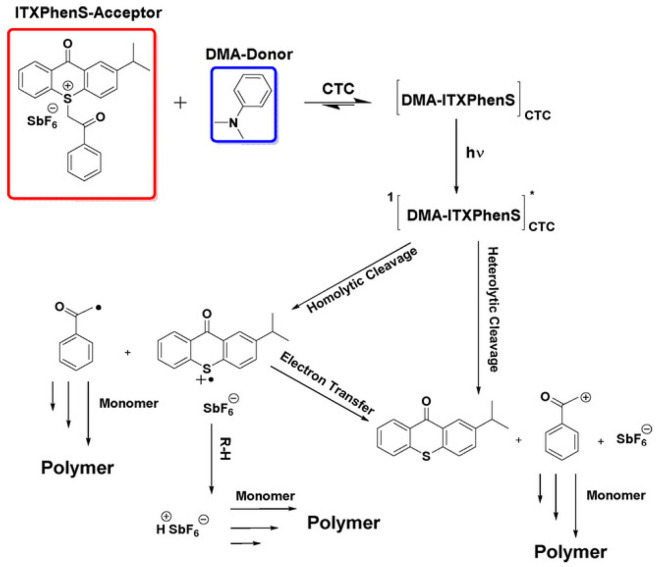
Mechanism of photoinitiation with the charge transfer complex based on ITXPhenS. Reproduced with permission of Ref. [287]. (* corresponds to the excited state).

As shown in Figure 30, absorbance of the ITXPhenS/DMA_CTC_ could be controlled by modifying the ITXPhenS to DMA ratio. By replacing a ITXPhenS/DMA_CTC_ 1:1 ratio for a 1:2 ratio, a redshift of the absorbance could be obtained. Thus, if an absorption band ranging between 425–500 nm was determined for the ITXPhenS/DMA_CTC_ 1:1 ratio, this absorption band was shifted to 500–625 nm for the ITXPhenS/DMA_CTC_ 1:2 ratio. Noticeably, the possibility to form CTCs with other electron-rich amines was examined such as triethylamine, triphenylamine and pyridine. However, the formation of CTC was not detected with these different amines. The different ITXPhenS/DMA_CTC_ proved to be versatile photoinitiators since photopolymerization experiments could be carried out at 350, 450 nm and even under sunlight. Using the sun as the light source, excellent monomer conversions could be obtained using isobutyl vinyl ether (IBVE) or methyl methacrylate (MMA) as the monomers, and a concentration of (5 × 10^−2^ M) for the CTC. Comparison of the polymerization profiles obtained during the photopolymerization of the difunctional triethylene glycol divinylether (TEGDVE) and triethyleneglycol dimethacrylate (TEGDMA) revealed that much faster polymerizations could take place with TEGDVE than with TEGDMA. Considering that TEGDVE and TEGDMA, respectively, polymerize by means of a cationic and a free radical mechanism, the initiation mechanism of ITXPhenS/DMA_CTC_ was presumed to occur by means of a homolytic and a heterolytic cleavage of the phenacyl moiety in the excited state, followed by an electron transfer. as classically observed for sulfonium salts (see Figure 31).

**Figure 32:**
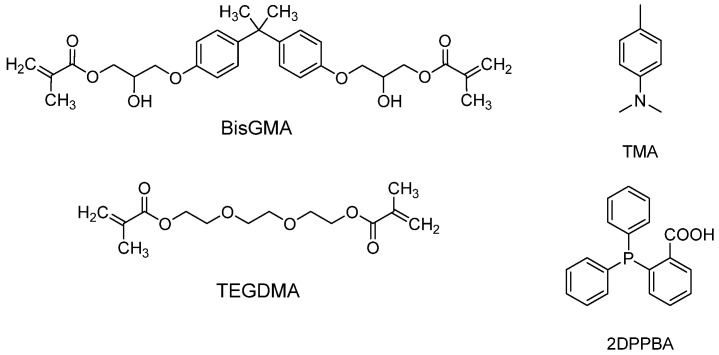
Chemical structures of BisGMA, TEGDMA, TMA and 2DPPBA.

More recently, the possibility of designing new CTCs with ITXPhenS was examined [170]. Notably, CTCs could be prepared using 4-*N*,*N*-Trimethylaniline (TMA) or 2-(diphenylphosphino)benzoic acid (2DPPBA) as the electron donors (see Figure 32). Interestingly, ITXPhenS/TMA_CTC_ (0.5%/0.25% *w*/*w*) and ITXPhenS/2DPPBA_CTC_ (1%/1% *w*/*w*) exhibited a dual curing mode since the FRP of a mixture of BisGMA/TEGDMA (70/30) could be photochemically and thermally initiated. As thermal initiator, the ITXPhenS/TMA_CTC_ could initiate the FRP of the BisGMA/TEGDMA blend with a maximum exothermic peak at approximately 120 °C whereas this maximal temperature increased up to 176 °C for the ITXPhenS/2DPPBA_CTC_. For comparison, thermal polymerization using ITXPhenS as the initiator could be initiated in almost similar conditions than that observed for the ITXPhenS/2DPPBA_CTC_ since the maximum exothermic peak was approximately 186 °C.

**Figure 33:**
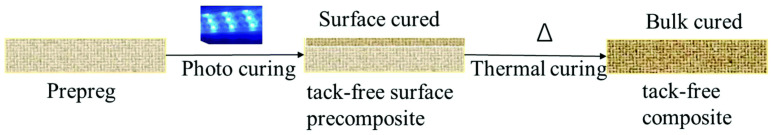
The strategy developed by Yagci and coworkers to prepare composites using first a photochemical curing followed in a second step by a thermal curing. Reproduced with permission of Ref. [287].

An excellent storage stability was determined for the two CTC-based resins since no modification of the polymerization profiles with that of freshly prepared resins was detected, even after 45 days of storage. Due to the photo/thermal dual curing abilities of the two CTCs, composites could be prepared. Using light, the surface of the composites could be efficiently polymerized, whereas the polymerization in bulk could be obtained by thermal curing (see Figure 33).

### 2.7. Fluorene-Based Sulfonium Salts

**Figure 34:**
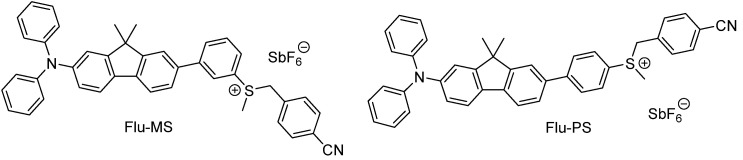
Chemical structures of Flu-MS and Flu-PS.

**Figure 35:**
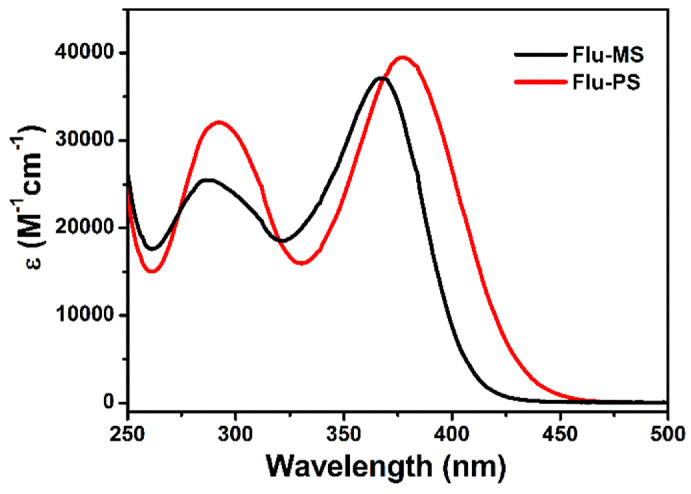
UV-visible absorption spectra of Flu-MS and Flu-PS in acetonitrile. Reproduced with permission of Ref. [288].

Different strategies were developed to redshift the absorption of the sulfonium salts, and an efficient strategy was proposed by using fluorene as a π-conjugated spacer [288]. Fluorene is an excellent electron-donating group that was notably used for the design of chromophores for solar cells [289,290,291,292,293,294,295,296,297,298,299], light emitters for OLEDs [300,301,302,303,304,305,306] and charge transport materials for OFETs [307,308,309,310,311,312,313,314,315] and as fluorescent probes [316,317,318,319,320,321,322,323]. The fluorene scaffold was notably used by Yagci and coworkers to design thioxanthone exhibiting a high molar extinction coefficient and a redshifted absorption compared with PI-PAG [324]. Influence of the substitution pattern was also examined, since Flu-MS and Flu-PS vary from each other by the position of the sulfonium moiety in *meta* and *para* positions, respectively (see Figure 34). As anticipated, Flu-PS exhibited a redshifted absorption compared with Flu-MS (378 nm for Flu-PS vs. 368 nm for Flu-MS), resulting from an extended conjugation in Flu-PS (see Figure 35). Jointly, a slight increase in the molar extinction coefficient could be evidenced for Flu-PS compared with Flu-MS (36 900 for Flu-MS vs. 39 400 M^−1^·cm^−1^). Irrespective of the substitution pattern, the two salts absorb up to 450 nm so that polymerization experiments could be carried out at 365, 385 and 405 nm using LEDs of low light intensity (2 mW/cm^2^). Photoacid generation quantum yields of 0.43 and 0.63 were, respectively, determined for Flu-PS and Flu-MS upon irradiation at 365 nm. In the case of Flu-MS, this value is higher than that previously reported for Mono-Ben (0.60 in Ref. [261] and 0.48 in Ref. [178]), not comprising a conjugated spacer. It therefore suggests that the elongation of the π-conjugation facilitates the dissociation of the S–C bond by stabilizing the intermediate radical cation.

In order to investigate the origin of the photoinduced electron transfer in Flu-MS and Flu-PS, the Gibbs free energy change (ΔGet) for an electron transfer occurring from the singlet excited state was determined for the two structures. Thus, values of −111.61 and −71.92 kJ/mol were, respectively, determined for Flu-MS and Flu-PS. These results are consistent with the photoacid generation quantum yields determined for the two structures, the highest photoacid generation quantum yield being obtained for the structure exhibiting the most negative ΔGet.

The higher reactivity of Flu-MS was confirmed during the CP of DVE-3, CHO, EPOX, the FRP of TMPTA, hybrid polymerization of an EPOX/TMPTA blend and the thiol-ene polymerization of trithiol/TMPTA performed upon irradiation at 365 nm and by using 1 wt% photoinitiators (I = 2 or 4 mW/cm^2^, see Table 8). During cationic polymerizations, the highest monomer conversions were obtained with DVE-3, with conversions higher than 98%. During the concomitant polymerization of an EPOX/TMPTA blend (50%/50% *w*/*w*), a higher TMPTA conversion was obtained when blended (70%) than during the FRP of TMPTA alone (56%), resulting from a synergetic effect between the CP of EPOX and the FRP of TMPTA. Among the most interesting results, extremely fast thiol-ene polymerization processes could be evidenced with Flu-MS and Flu-PS since the maximum conversions for TMPTA and trithiol could be obtained within 10 s.

Examination of the monomer conversions obtained for different monomers at longer wavelengths revealed the two sulfonium salts to be capable of maintaining a higher monomer conversion, even upon irradiation at 450 nm (See Table 9).

**Figure 36:**
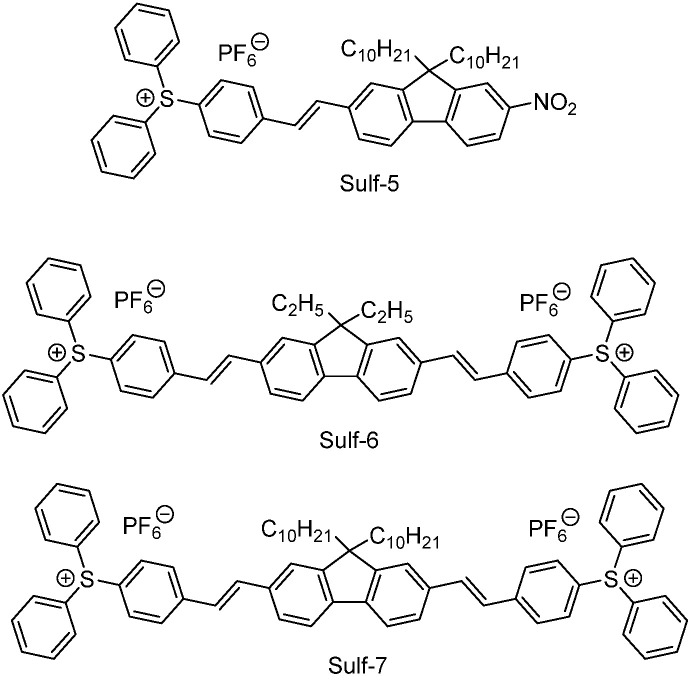
Chemical structures of fluorene-based photoinitiators developed by Belfield and coworkers.

The design of more extended structures was also examined by Belfield and coworkers who developed a series of two-photon photoinitiators based on fluorenes (see Figure 36) [256]. These structures were notably proposed as photoinitiators for two-photon 3D write-once read-many (WORM) optical data storage systems.

### 2.8. Anthracene-Based Sulfonium Salts

**Figure 37:**
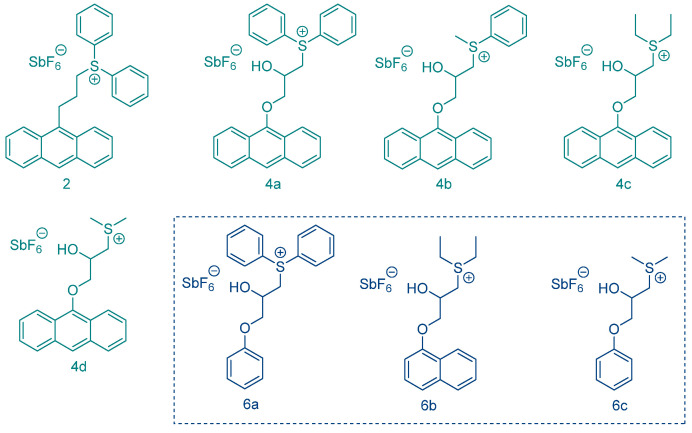
Chemical structures of anthracene-substituted sulfonium salts, different reference compounds and the epoxy monomer SU-8 and TPGE.

Sulfonium salts dramatically lack absorption above 320 nm, but their spectral response can be significantly improved by the covalent linkage of the sulfonium group to anthracene (see Figure 37) [325]. Anthracene is an excellent photosensitizer that was used numerous times in photopolymerization [34,69,326,327,328,329,330,331,332,333,334]. Using this strategy, absorption of the different sulfonium salts could range between 330 and 410 nm, benefiting from the strong absorption of anthracene in this spectral range. Consequently, anthracene could act as a photosensitizer for the sulfonium salts, enabling the generation of mono-component photoinitiating systems. For comparison, a series of sulfonium salts were designed with a phenyl group (see compounds **6a**–**6c**).

**Scheme 6:**
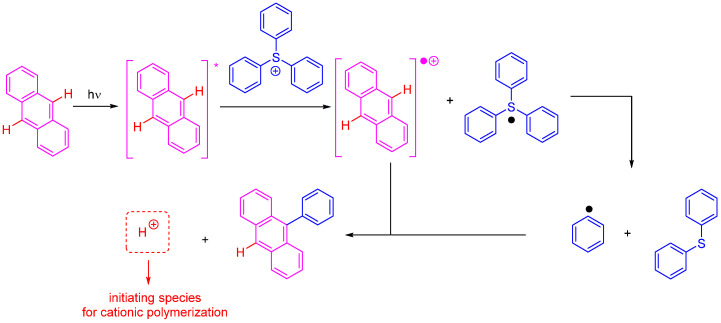
Mechanism of formation of 9-phenylanthracene starting from anthracene and triphenylsulfonium. (* corresponds to the excited state).

Photoinitiating ability of the different structures was investigated during the cationic polymerization of a mixture of three monomers comprising *tris*(4-hydroxyphenyl) methane triglycidyl ether (TPGE), SU-8 and EPOX upon irradiation with a medium pressure mercury lamp (500 mJ/cm^2^) and using a 2.5 wt% photoinitiator. Interestingly, during the CP of the blend of three monomers (SU-8, TPGE and EPOX), **2** and **4a** showed a photosensitivity six times higher than that of **4b,** bearing only one aromatic ring on the sulfonium side. In the case of the alkyl-substituted sulfonium salts, **4c** and **4d**, no photoinitiating activity was detected. Comparison with a benchmark photoinitiator composed of a mixture of diphenyl-4-thiophenoxyphenylsulfonium hexafluoroantimonate and *bis*[4-(diphenylsulfonio)phenyl]sulfide *bis*-hexafluoroantimonate (GR61) revealed **2** and **4a** (and even **4b**) to exhibit a higher photosensitivity than the benchmark photoinitiator GR61 when sensitized by 9-methylanthracene, evidencing the interest of the intramolecular approach and the covalent linkage of the aryl-substituted sulfonium groups by the anthracene chromophore. Photoacid generation quantum yields ϕ_H+_ of **2** and **4b** in acetonitrile using a merocyanine indicator dye furnished values of 0.32 and 0.21, respectively, consistent with the order of photoactivity determined during the photopolymerization experiments. Examination of the photoproducts formed during photolysis of the anthracene/GR61 combination revealed phenylated anthracene to be formed in 55% yield, with 9-phenylanthracene as the main side-product (73%). The formation of 9-phenylanthracene was assigned to a photoinduced electron transfer between the excited anthracene (in its singlet excited state) and the electrodeficient sulfonium salt [163,335]. Then, by an in-cage combination of an aryl radical and the radical cation of anthracene, 9-phenylanthracene can be formed subsequent to a proton elimination (see Scheme 6).

Based on the previous work of Sharma and coworkers, the authors suggested the good polymerization efficiency of **2** and **4a** is unlikely to arise from an inefficient back electron transfer [336]. Indeed, back electron transfer in photopolymerization is one of the major drawbacks of the photosensitization approach [337,338,339]. Although well-known, this undesired back electron transfer cannot be anticipated. More likely, the occurrence of an efficient electron-transfer induced concerted bond cleavage and a cation radical/radical coupling mechanism was suggested as a plausible explanation to support the polymerization efficiency of **2** and **4a**, in light of the previous work carried out by Luss and their coworkers on intramolecular photoinduced rearrangements of *para*-cyanobenzylated anthracenes [166]. Photolysis experiments performed in the solution confirmed the good reactivity of **4a**, with a decrease in the absorption intensity of 55% after 30 min of irradiation in acetonitrile. Fluorescence experiments revealed that **2**, **4a** and **4b** exhibit a low fluorescence intensity, indicative of an intramolecular electron transfer from the excited singlet state of anthracene to the sulfonium group. Conversely, fluorescence quenching of anthracene was not detected for **4c** and **4d**, evidencing the lack of photoinduced electron transfer. Using the Rehm–Weller equation, the free energy change ΔG for **4a**, **4b** and **4d** could be determined. ΔG values of −107, −49 and −20 kJ/mol were determined, confirming that the thermodynamic was unfavorable for an intramolecular electron transfer for **4d**.

### 2.9. Coumarin-Based Sulfonium Salts

**Figure 38:**
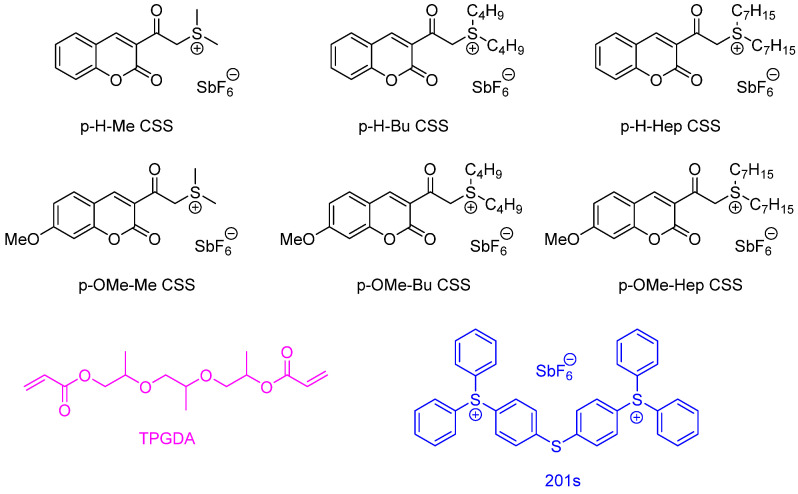
Chemical structures of coumarin-based sulfonium salts, TPGDA and the sulfonium salt 201s.

**Figure 39:**
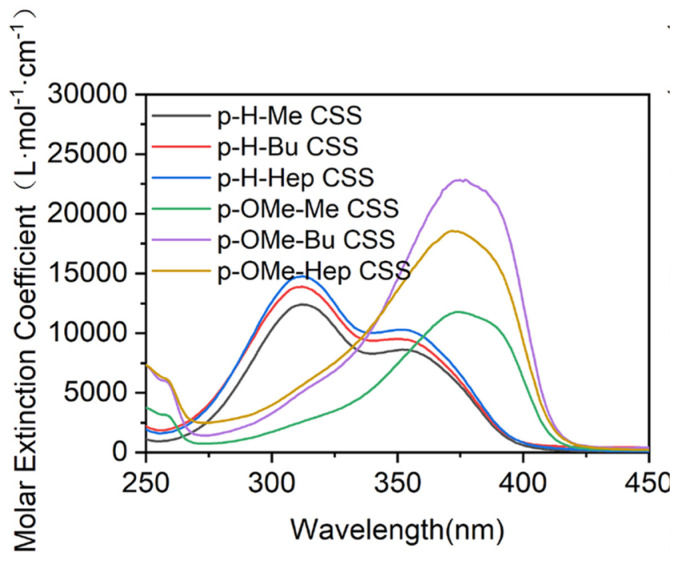
UV-visible absorption spectra of coumarin-based sulfonium salts in acetonitrile. Reproduced with permission from Ref. [355].

Coumarins are biosourced or bioinspired structures [340,341,342] that have been extensively used for the design of photoinitiating systems [343,344,345,346,347,348]. Coumarins, by their absorptions located in the near-UV-visible range, are appropriate photoinitiators for photopolymerization experiments performed at 405 nm, which is the wavelength used in 3D printers [349,350,351,352,353,354]. In 2023, a series of coumarin-substituted sulfonium salts bearing long alkyl chains for solubility was proposed by Liu and coworkers and examined in cationic, free radical and hybrid photopolymerizations (see Figure 38) [355]. From the absorption viewpoint, the introduction of a methoxy group in p-OMe-Me CSS, p-OMe-Bu CSS and p-OMe-Hep CSS contributed to the redshift of the absorption of the different dyes and increase in the molar extinction coefficient by favoring the electronic delocalization. Thus, if an absorption maximum at approximately 380–390 nm was found for p-OMe-Me CSS, p-OMe-Bu CSS and p-OMe-Hep CSS in acetonitrile, an absorption maxima at 300–320 nm was determined for p-H-Me CSS, p-H-Bu CSS and p-H-Hep CSS (see Figure 39). The crucial role of the electron-donating methoxy group on the optical properties of the sulfonium salts was thus demonstrated. The beneficial effect of the methoxy group was also determined during the steady-state photolysis experiments performed upon irradiation at 365 and 405 nm. Thus, all seven substituted sulfonium salts could decompose faster than the unsubstituted ones, resulting in their redshifted absorptions better fitting with the emission of LEDs.

**Figure 40:**
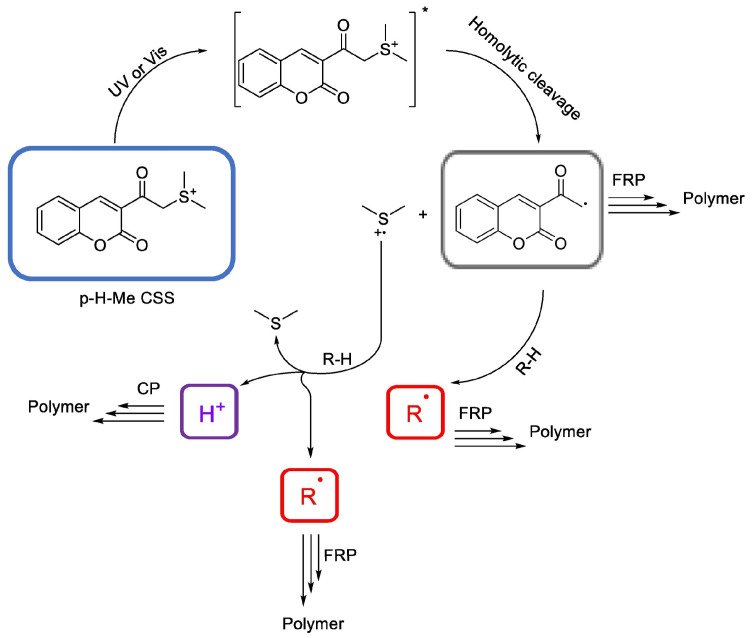
Mechanism of photoinitiation with coumarin-based sulfonium salts. Reproduced with permission from Ref. [355]. (* corresponds to the excited state).

By ESR experiments, formation of the acetyl coumarin radical and dimethyl sulfide radical cations could be demonstrated, resulting from the homolytic cleavage of the C–S bond. By GC-MS analysis, formation of acetylcoumarin could also be evidenced, confirming the ESR results. In light of these results, the mechanism of photoinitiation could be determined and the mechanism that was proposed by the authors is depicted in Figure 40.

The investigation of their photoinitiating abilities during the CP of EPOX and the FRP of TMPTA revealed the good adequation between molar extinction at the irradiation wavelength and monomer conversions (see Table 10). Control experiments were carried out with *bis*[4-(diphenylsulfonio)phenyl] sulfide *bis*(hexafluoroantimonate) (201s) for the CP of EPOX and ITX for the FRP of TMPTA. Photopolymerization tests were carried out at 365 nm (I = 70 mW/cm^2^) and 405 nm (I = 70 mW/cm^2^), using a concentration of 5.27 × 10^−5^ mol/g photoinitiators.

Interestingly, by varying the length of the alkyl chains on the sulfonium group from one to seven carbons, an optimal situation was found for p-H-Bu CSS and p-OMe-Bu CSS, both bearing butyl chains. As can be seen in Table 10, the best monomer conversions obtained at 365 and 405 nm during the CP of EPOX and the FRP of TMPTA were obtained for these two photoinitiators. These results are remarkable considering that monomer conversions outperforming that obtained with 201s and ITX could be determined.

**Figure 41:**
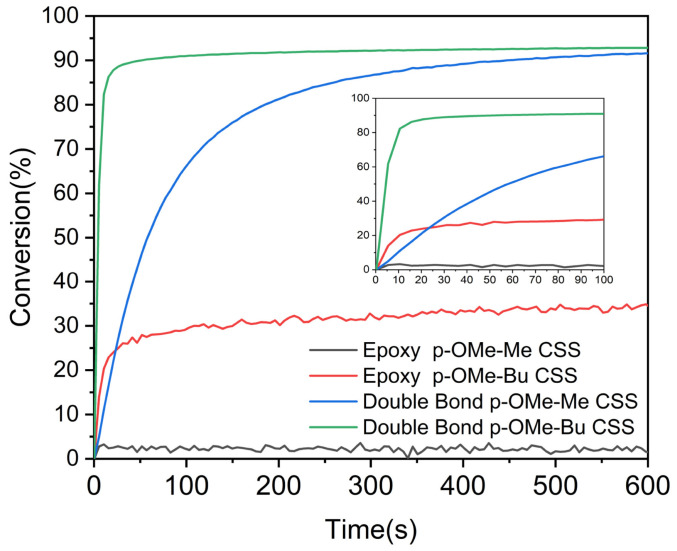
Hybrid polymerization of TPGDA and EPOX using p-OMe-Me CSS and p-OMe-Bu CSS upon irradiation at 365 nm (I = 70 mW/cm^2^, sulfonium salt concentration: 5.27 × 10^−5^ mol/g). Reproduced with permission from Ref. [355].

Considering the high monomer conversions obtained during the cationic polymerization and the free radical polymerization, hybrid polymerization of tripropylene glycol diacrylate (TPGDA) and EPOX was investigated. Noticeably, the FRP of TPGDA proceeded faster than the CP of EPOX, the radicals being directly formed upon photoexcitation of the photoinitiators, contrarily to the cation species that are formed in successive reactions (see Figure 41). This point was especially evidenced with p-OMe-Me CSS.

## 3. Conclusions

Sulfonium salts are efficient photoinitiators suffering from a weak absorption in the visible range. Over the years, this issue was addressed by connecting the sulfonium groups to different chromophores such as Bodipy, 1,3,5-triphenyl-2-pyrazolines, triphenylamines, fluorenes, anthracene or coumarins. The choice of these chromophores for generating monocomponent photoinitiating systems is directly related to the recent advances in the design of Type II photoinitiating systems where these structures have emerged as promising scaffolds. As specificities, these different chromophore-bonded sulfonium salts have been obtained by means of multistep syntheses. If the resulting structures are costly, the efficiency of the intramolecular electron transfer vs. the intermolecular one has been clearly demonstrated, evidencing the pertinence of this approach. The formation of charge transfer complexes of sulfonium salts with different electron-rich amines is an interesting approach enabling the minimization of the synthetic efforts. Indeed, such structures can be directly prepared from commercially available sulfonium salts and aromatic amines. Among the most recent advances, up-conversion nanoparticles have been used to promote the photocleavage of sulfonium salts while exciting in the near-infrared range, i.e., in safe irradiation conditions. As other advantages of these mono-component systems, chromophores bonded to the sulfonium group greatly contributed to the generation of sulfonium salts soluble in resins, which constitutes a major drawback of most of the benchmark sulfonium salts. At present, only a few sulfonium salts showed sufficient reactivity to initiate polymerization processes under sunlight. Furthermore, such structures would be of interest for numerous outdoor applications. At present, water-soluble sulfonium salts activable in the visible range have not been reported yet, to the best of our knowledge. In addition, photopolymerization in water could become possible, enabling polymerization in green conditions. Sensitivity to sunlight and the development of water-soluble sulfonium salts will certainly be the focus of intense works in the coming years.

## Figures and Tables

**Table 1:** Comparisons between sulfonium-bounded sulfonium salts and the other sulfonium salts that are commercially available and used in intermolecular sensitization systems.

Parameters	Monocomponent Sulfonium Salts	Other Sulfonium Salts
Cost/synthesis	Compounds are often prepared in multistep syntheses rendering these compounds expensive.	Numerous sulfonium salts are commercially available and can be purchased at low cost.
Environmental impact	Most synthetic dyes are often prepared in an organic solvent. Purification by column chromatography is also required.	The same comments can be performed concerning benchmark sulfonium salts.
Photochemical stability	Chromophore-substituted sulfonium salts are stable compounds. Therefore, their photochemical stability is good.	Sulfonium salts are highly stable, notably when comprising aromatic rings.
Absorption range	Chromophores bounded to sulfonium salts exhibit a broad absorption and their absorption spectra can be finely tuned from the near-UV visible range up to the near-infrared range	Benchmark sulfonium salts exhibit a UV-centered absorption, with no absorption in the visible range.
Photoinitiating ability	Chromophores are ideal candidates for photoinitiation in the visible range and even in the near-infrared region. Ability of chromophores to release or accept electrons is well-documented in the literature.	Good photoinitiating ability of sulfonium salts in the UV range
Availability	Availability can be restricted, especially when multistep syntheses are used. Expensive compounds, which can limit their scope of applications.	Easy availability. Benchmark photoinitiators can be purchased in the Kg scale at low cost. Numerous applications of benchmark sulfonium salts in industry.

**Table 2:** Quantum yields of photoacid generation (ϕ_H+_) upon irradiation with different LEDs.

	365 nm	385 nm	395 nm	405 nm	425 nm
PI-CF3	0.60	0.56	0.61	0.59	0.31
PI-H	0.62	0.59	0.62	0.63	0.26
PI-EtO	0.65	0.61	0.64	0.46	0.20

**Table 3:** EPOX conversions (%) determined by photo-DSC upon irradiation at different wavelengths and by using 1 wt% photoinitiator.

	365 nm	385 nm	395 nm	405 nm	425 nm
PI-CF3	49.15	50.63	52.66	55.18	51.87
PI-H	52.84	54.06	56.37	59.74	40.25
PI-EtO	55.06	56.47	58.82	61.97	49.57
PI-6992M	59.31	43.63	-	-	-

**Table 4:** TMPTA conversions (%) determined by photo-DSC upon irradiation at different wavelengths and by using 1 wt% photoinitiator.

	365 nm	385 nm	395 nm	405 nm
PI-CF3	43.1	44.9	41.3	35.3
PI-H	50.9	48.9	45.5	42.4
OXE-02	65.6	61.5	44.4	27.0

**Table 5:** Polymerization thresholds (mW) determined during the polymerization of Araldite CY179MA epoxide resins upon irradiation at 710 nm.

Initiator	λ = 710 nm
BSB-S2	2.4
CD1012/ITX	44
CD1012	212
TPS	>317 ^a^
DPI-DMAS	>317 ^a^

^a^: no polymerization detected at the maximum power, i.e., 317 mW.

**Table 6:** Photophysical properties of PAG1-PAG6 determined in acetonitrile.

Initiator	λ_max_ (nm)	ε_max_ (M^−1^·cm^−1^)	ϕ_H+_ ^a^	δ_max_ (GM)
PAG1	346	36,100	0.10	73 (760 nm)
PAG2	324	26,000	0.24	68 (710 nm)
PAG3	395	34,300	0.05	643 (870 nm)
PAG4	381	23,700	0.44	650 (800 nm)
PAG5	400	34,400	0.31	680 (880 nm)
PAG6	380	25,200	0.50	648 (800 nm)

^a^ determined at 405 nm.

**Table 7:** Photophysical properties of a series of multibranch sulfonium salts.

Compound	λ_abs_ (nm)	ε_max_ (M^−1^·cm^−1^)	ϕ_H+_ ^a^	ϕ_H+_ ^b^	δ780 nm (GM)	ϕ_H+_
Pre-Para	394	53,200	-	-	-	-
Mono-Para	404	40,600	0.002	0.02	528	1.0
Bi-Para	413	37,000	0.004	0.04	816	3.2
Pre-Meta	385	37,000	-	-	-	-
Mono-Meta	392	32,000	0.20	0.41	234	46.8
Bi-Meta	399	30,800	0.40	0.49	745	298

^a^ Quantum yield of photoacid generation at 405 nm in acetonitrile. ^b^ Quantum yield of photoacid generation at 405 nm in dichloromethane.

**Table 8:** Monomer conversions determined with Flu-MS and Flu-PS using different monomers or monomer blends upon irradiation at 365 nm using 1 wt% photoinitiator.

Monomer	Photoinitiator	Intensity (mW/cm^2^)	Conversion (%)
DVE-3	Flu-MS	2	98
DVE-3	Flu-PS	2	99
CHO	Flu-MS	2	84.5
CHO	Flu-PS	2	79
EPOX	Flu-MS	4	70
EPOX	Flu-PS	4	59
TMPTA	Flu-MS	4	58
TMPTA	Flu-PS	4	61
EPOX/TMPTA	Flu-MS	2	39/71
EPOX/TMPTA	Flu-PS	2	43/71
trithiol/TMPTA	Flu-MS	2	50/82
trithiol/TMPTA	Flu-PS	2	47/85

**Table 9:** Conversions of EPOX, CHO, TMPTA and EPOX/TMPTA at different irradiation wavelengths and intensities using Flu-MS or Flu-PS (1 wt%).

Monomers	PIs	Light Source	Intensity (mW/cm^2^)	Conversion (%)
EPOX	Flu-MS	385 nm	4	65.6
EPOX	Flu-MS	405 nm	4	73.0
EPOX	Flu-MS	425 nm	4	63.2
EPOX	Flu-PS	385 nm	4	63.3
EPOX	Flu-PS	405 nm	4	68.6
EPOX	Flu-PS	425 nm	4	64.3
CHO	Flu-MS	385 nm	2	81.1
CHO	Flu-MS	405 nm	2	64.3
CHO	Flu-MS	450 nm	16	65.0
CHO	Flu-PS	385 nm	2	76.3
CHO	Flu-PS	405 nm	2	76.9
CHO	Flu-PS	450 nm	16	85.4
TMPTA	Flu-MS	385 nm	4	48.5
TMPTA	Flu-MS	405 nm	4	42.8
TMPTA	Flu-PS	385 nm	4	60.8
TMPTA	Flu-PS	405 nm	4	56.2
EPOX/TMPTA	Flu-MS	365 nm	2	22.8/5.3
EPOX/TMPTA	Flu-PS	365 nm	2	13.1/13.3

**Table 10:** Monomer conversions (%) obtained during the CP of EPOX and the FRP of TMPTA upon irradiation at 365 and 405 nm (I = 70 mW/cm^2^) with LEDs.

365 nm	p-H-Me CSS	p-H-Bu CSS	p-H-Hep CSS	p-OMe-Me CSS	p-OMe-Bu CSS	p-OMe-Hep CSS	
EPOX	18	64	37	18	55	32	55 (201s)
TMPTA	44	69	54	48	62	54	68 (ITX)
405 nm	p-H-Me CSS	p-H-Bu CSS	p-H-Hep CSS	p-OMe-Me CSS	p-OMe-Bu CSS	p-OMe-Hep CSS	201s
EPOX	12	53	44	15	69	45	12 (201s)
TMPTA	40	58	43	39	60	52	65 (ITX)

## Data Availability

No data available.
